# An ultra-compact and high isolated 8 × 8 MIMO antenna system for 5G NR-n46 and n79 band applications

**DOI:** 10.1038/s41598-026-43426-7

**Published:** 2026-03-07

**Authors:** Brijesh Mishra, R. Sethumadhavi, Sweta Singh, Saiyed Salim Sayeed, Aditya Kumar Singh, Amrees Pandey, Tanweer Ali

**Affiliations:** 1 Department of Electronics and Communication Engineering, School of Engineering and Technology, CMR University, Bengaluru, Karnataka India; 2 School of Computer Science and Engineering, REVA University, Rukmini Knowledge Park, Yelahanka, Bengaluru, Sathanur, Kattigenahalli, Karnataka India; 3 United University Prayagraj, Prayagraj, Uttar Pradesh India; 4 Institute of Technology and Management, Maharajganj, Uttar Pradesh India; 5 Department of Interdisciplinary Courses in Engineering, Chitkara University Institute of Engineering and Technology, Chitkara University, Chitkara, Punjab India; 6 Department of ECE, Shambhunath Institute of Engineering and Technology (SIET), Prayagraj, India; 7 Manipal Institute of Technology, Manipal Academy of Higher Education, Manipal, India

**Keywords:** MIMO antenna, 5G NR bands, Compact size, Diversity and spatial multiplexing, Annular ring, Licensed assisted access (LAA), Energy science and technology, Engineering

## Abstract

This article presents an ultra-compact (1.02λ × 1.02λ mm^2^) and highly isolated 8-port MIMO antenna designed for NR-n46 and n79 bands, as well as licensed assisted access (LAA). A systematic study was performed to choose an optimal antenna (Design-3) among all designs (Design-1, Design-2, and Design-3) after systematic study (parametric study and circuit theory analysis) of Ref. design-1, Ref. design-2, Ref. design-3 and Ref. design-4. An optimal and proposed antenna geometry consists of two orthogonal radiators on the top and a novel ground plane (rectangular ring, centered annular ring and plus shaped slot) at the bottom of each corner of the dielectric substrate to create a perfectly matched 8-port antenna. The proposed antenna demonstrates a wideband frequency operation of 700 MHz within the 4.75–5.45 GHz range, specifically in the sub-6 GHz 5G band. It resonates at 5.2 GHz, achieving an isolation of 33 dB, a gain of 4.7 dB, and a radiation efficiency of 92.5%. The MIMO characteristics, including ECC, DG, TARC, MEG, and CCL, were evaluated and found to be within acceptable parameters. The antenna was fabricated, tested in a laboratory setting, and its performance was validated against simulated results.

## Introduction

The introduction of next-generation mobile networks such as 5G and 6G has significantly increased the demand for high-speed wireless communication systems. For greater capacity, faster data rates, and reliable connectivity, the 5G New Radio (NR) standard is necessary, particularly in sub-6 GHz frequency bands, such as n46 (5.15–5.925 GHz) and n79 (4.4–5.0 GHz). In release 16 of Third Generation Partnership Project (3GPP), 5G NR was extended to prioritize unlicensed band to 5150–5925 MHz for Wi-Fi and LTE-based LAA applications. To accomplish these objectives, multiple-input multiple-output (MIMO) technology is frequently utilized because of its capacity to boost signal robustness, increase spectral efficiency, and facilitate huge communication^1^. The 5G New Radio (NR) n46 and n79 band frequencies are critical spectrum resources that enable high-capacity, high-speed wireless communication, especially in advanced MIMO-based antenna systems^2^. In particular, n46 lies within the 5.1–5.3 GHz spectrum and is classified as an unlicensed band, enabling its combination with Licensed Assisted Access (LAA) to increase capacity by aggregating with licensed spectrum to enhance downlink throughput, without requiring exclusive spectrum ownership. The n79 band operates within the 4.4–5 GHz range and represents a licensed mid-band resource, offering the optimal balance between low-latency coverage and capacity, making it highly suitable for densely populated urban areas and massive device connectivity^3^.

With an 8-port MIMO antenna configuration, both n46 and n79 can be operated concurrently, enabling multi-band, multi-layer transmission that delivers significantly improved spectral efficiency, throughput, and reliability through spatial multiplexing and diversity. Combining LAA with an 8-port MIMO system allows network operators to pair licensed mid-band spectrum (n79) with unlicensed spectrum (n46), supporting enhanced mobile broadband (eMBB), ultra-reliable low-latency communications (URLLC), and capacity offloading in high-traffic environments. These systems are particularly valuable in locations requiring high data rates and predictable quality of service, such as stadiums, transport hubs, and smart city infrastructures, where higher-order MIMO spatial diversity is complemented by the synergy of licensed–unlicensed aggregation^4^.

Large-element MIMO antennas are essential for contemporary wireless communication systems, particularly for 5G and beyond. The main benefit of a high-element MIMO system is its capacity to use spatial variety, which raises data speed, improves signal dependability, and increases spectral efficiency^5^. The principle of superposition states that beamforming and spatial multiplexing are made possible by the complex electromagnetic field patterns produced by several radiating devices. Beamforming is a technology that enhances the performance of a network by minimizing the interference and maximizing signal power through a number of antennas in order to concentrate the energy towards a certain direction^6^. The Shannon-Hartley theorem states that high data rates can be attained by raising either the bandwidth, or the signal-to-noise ratio (SNR). Using methods such as maximum ratio combining (MRC) and eigen beamforming, large-element MIMO antennas increase the SNR by lowering multipath fading and offer greater diversity advantages^7^.

In addition, MIMO systems with more elements use array gain to reduce the effects of path loss. The received power is proportional to the product of the transmitting and receiving antenna gains according to the Friis transmission equation. More antenna elements improve the system’s effective gain and guarantee better signal reception, particularly in crowded cities with many obstructions^8^. Several MIMO antenna such as high isolated 8-port MIMO antenna for next generation communications and internet of things (IOT) applications^9^, enhanced spectral efficiency 8 × 4 antenna for 6G applications^10^, self-decoupled 8-port antenna with low SAR (0.85 W/Kg) for 5G applications^11^, 4-port metamaterial superstrate based MIMO antenna for Wi-Fi and WIMAX applications^12^, 4-port substrate integrated coaxial line (SICL) based MIMO antenna for 5G/6G applications^13^ and in context of wireless communication, machine learning algorithm based approach for MIMO antenna has been seen in several reports^14,15^. MIMO antennas still have a number of drawbacks, though, such as poor isolation, large size that makes it difficult to integrate into portable devices, restricted sub-6 GHz band frequency coverage, high element correlation, low gain, and constraints on the number of antenna elements per board^5,16^.

An ultra-compact 8-port MIMO antenna for 5G-NR applications is chosen for this work because 8-port MIMO antenna enables high spatial multiplexing as compared to multiport (lower than 8-port i.e. 2, 4 & 6 port) MIMO antennas. The study presented in this paper demonstrates excellent performance matrices of 8-port MIMO antenna and encounters the constraints of a MIMO antenna, as mentioned in the previous paragraph. This work investigates the design, modeling, and performance evaluation of an 8-port MIMO antenna system suitable for the 5G NR-n46 and n79 bands. The proposed 8-port antenna structure is designed over an RT/DUORID (5870 tm) substrate and is novel in terms of the following factors:

- Unique antenna geometry (two antenna elements at each corner of substrate) with novel decoupler structure.
- An equivalent circuit model of the proposed antenna has been developed for deeper insight into its operating mechanism and validating its resonating performance.
- It is compact (60 × 60 mm^2^) and can be easily integrated into portable devices.
- A high isolation (33 dB) and perfect impedance matching (50 Ω) were obtained.
- Large frequency coverage for n46 and n79 bands.
- High antenna gain (4.7 dB) and efficiency (92.5%).
- Excellent diversity parameters; ECC (4.6×${10}^{-6}$), DG, TARC, MEG and CCL (0.04).
- Applicable and beneficial for carrier aggregation (CA) applications.
- The prototype supports diversity as well as spatial multiplexing.

This paper is divided into several sections, such as antenna development stages, antenna geometry, s-parameters, far-field study, MIMO characteristics, state-of-art, and conclusions for detailed study in the foregoing sections.

## Antenna development stages

### Development of single and two element MIMO antenna

A systematic investigation of the reflection and transmission coefficients using four reference designs (Ref. design-1, Ref. design-2, Ref. design-3 and Ref. design-4) was performed to determine the base antenna (Ref. design-4) for MIMO antenna implementation. The reference designs are intermediate designs used for the study and final selection of an optimal antenna for designing an 8-port MIMO antenna. The specification of the proposed antenna (substrate: RT/DUORID (5870 tm), dielectric constant (${\epsilon}_{r}$): 2.33, loss tangent (tan δ) = 0.009, substrate height: 1.6 mm, resonating frequency: 5.2 GHz) is fixed first and thereafter physical dimensions of Ref. design-1are calculated according to Eqs. (1–5) reported in^17^ and are given below:

1 $${\rm Y}=\frac{c}{2{f}_{o}}\sqrt{\frac{2}{{\epsilon}_{e}+1}}$$

2 $${\epsilon}_{e}=\frac{{\epsilon}_{r}+1}{2}+\frac{{\epsilon}_{r}-1}{2}{\left(1+\frac{12\mathrm{h}}{\mathrm{X}}\right)}^{-\frac{1}{2}}$$

 

3 $$\Delta\:{\rm Y}=0.412\mathrm{h}\times\frac{\left({\epsilon}_{e}+0.30\right)(\frac{X}{h}+0.264)}{\left({\epsilon}_{e}-0.258\right)(\frac{X}{h}+0.8)}$$

 

4 $${\rm Ye}=Y+2{\Delta}Y$$

5 $${Z}_{0}=\frac{120\pi}{\sqrt{{\epsilon}_{\mathrm{e}}}}{\left[\frac{X}{h}+1.393+0.667\mathrm{ln}\left(\frac{X}{h}+1.444\right)\right]}^{-1}$$

Where Y & X = length and width of radiator element of Ref. design-1, ${\epsilon}_{e}$ = effective dielectric constant, ΔY = residual length, ${Z}_{0}$ = characteristics impedance, Ye-effective length of radiator element.

**Fig. 1:**
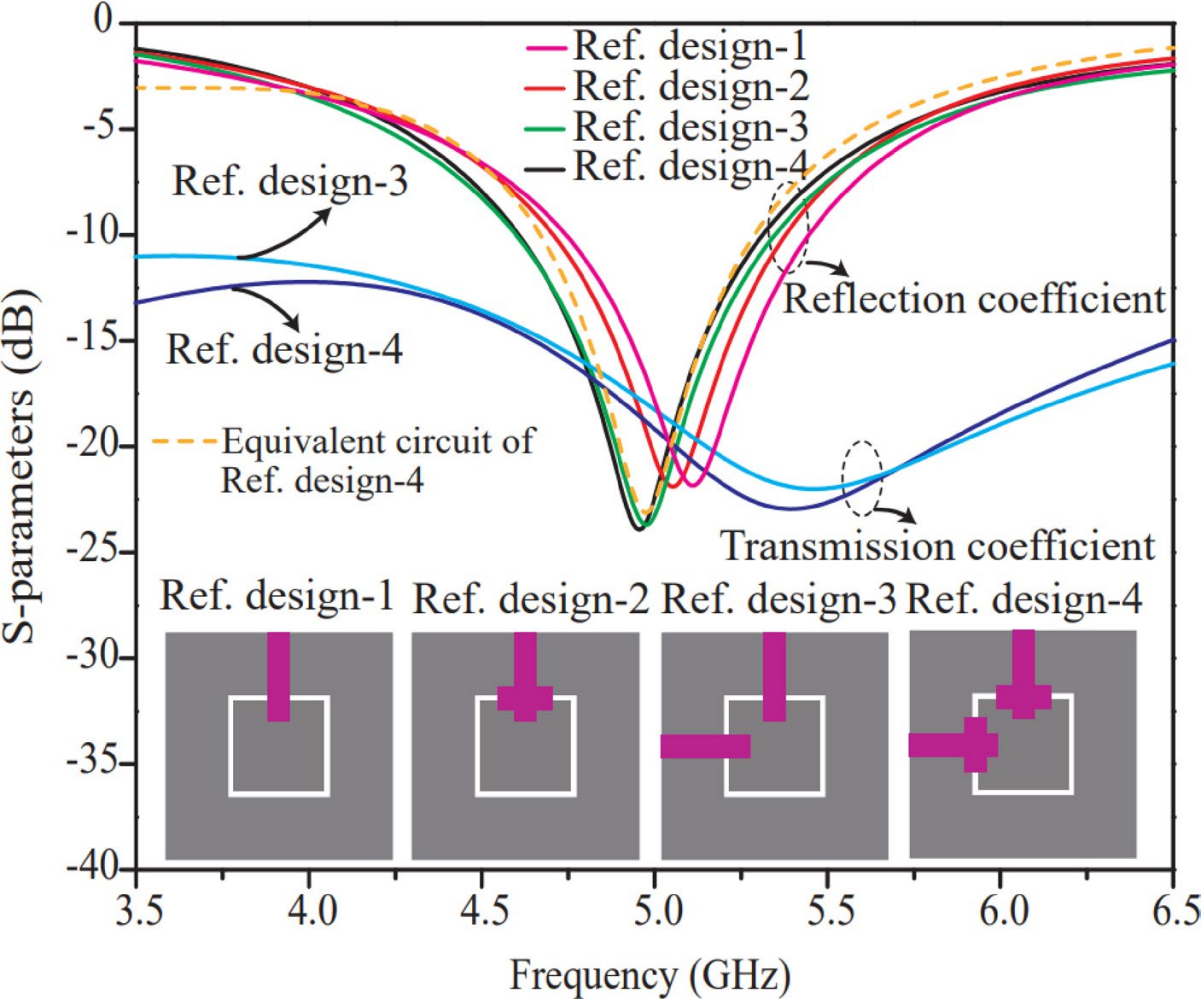
S-parameter curves of distinctive designs (Ref. design-1, Ref. design-2, Ref. design-3 and Ref. design-4 (proposed)).

A graph of S-parameters (dB) versus frequency (GHz) for four distinct reference designs labeled in Ref. design-1, Ref. design-2, Ref. design-3, and Ref. design-4 is displayed in Fig. 1. Both the reflection and transmission coefficients are displayed in the graph, emphasizing how the distinctive designs perform differently. The four reference designs are represented graphically beneath the graph, showing various geometrical arrangements of the resonant structures. From Fig. 1, it is observed that the single-port antenna design (Ref. design-1 and Ref. design-2) resonates at approximately the same frequency that is 5.1 GHz with the same bandwidth, but two-element MIMO of Ref. design-2 performs better owing to its high isolation. Therefore, Ref. design-4 was chosen as the optimal antenna for 8-port antenna development and further discussion.

### Equivalent lumped element circuit diagram

An equivalent circuit diagram of a two-port antenna (Ref. design-4) is presented, validated, and discussed in this section for further implementation of an eight-port antenna design. A cavity model-based circuit theory approach is used to analyze the equivalent circuit diagram of the antenna. This technique offers an R-L-C equivalent circuit after considering the patch antenna as a resonant cavity. The patch antenna behaves as a resonant cavity because the radiating and ground planes are separated by a dielectric substrate, wherein the electric field stores electric energy in the substrate region, resulting in capacitor-like behavior (C), and the magnetic field stores magnetic energy in this region, which acts as an inductor (L). Radiation, conductor, and dielectric losses dissipate energy, which makes it a resistive element (R).

**Fig. 2:**
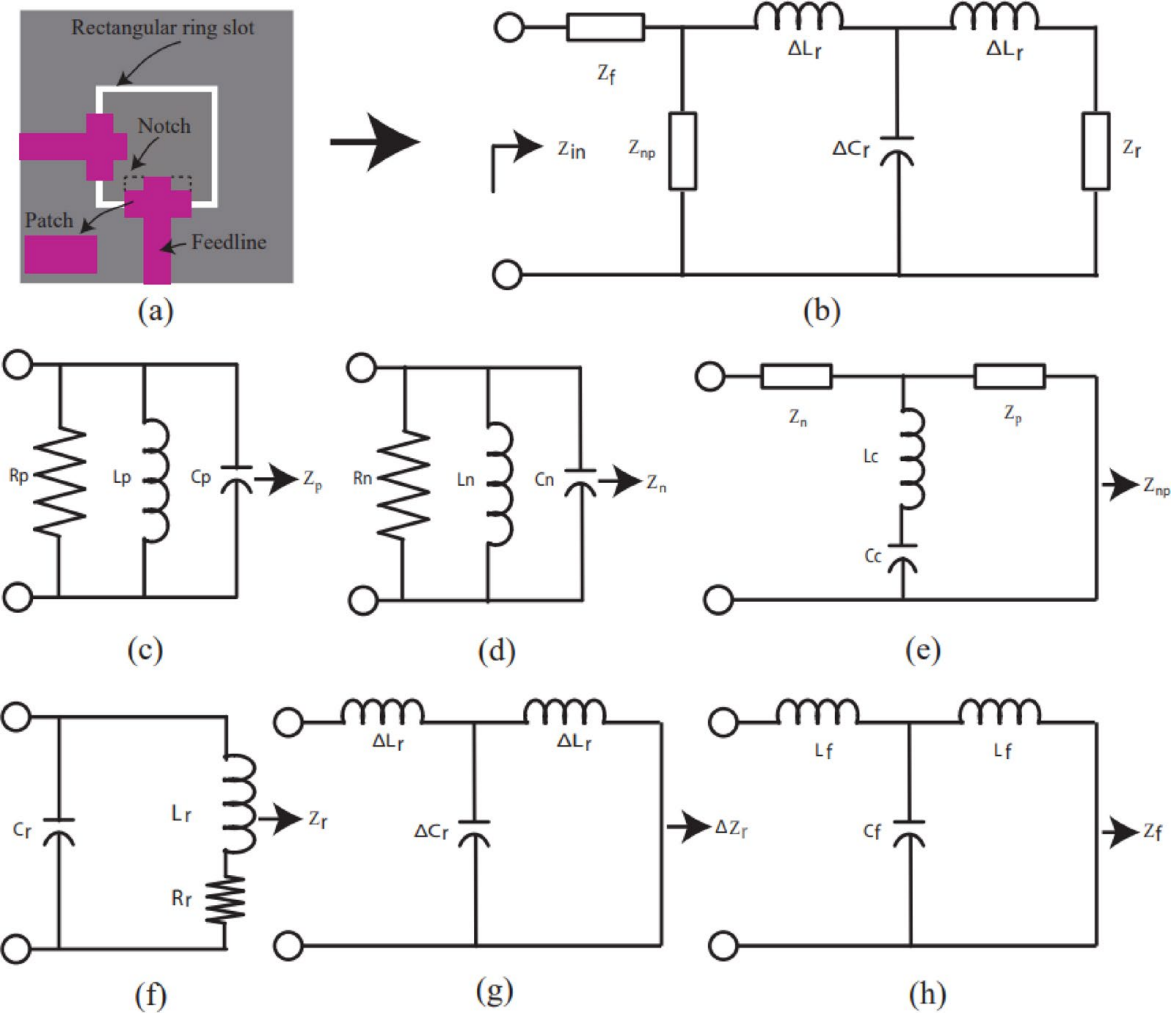
(**a**) Two port antenna geometry (**b**) Circuit diagram of the proposed antenna (**c**) Rectangular patch antenna circuit (**d**) Notch circuit (**e**) Notch loaded patch antenna circuit (**f**) Rectangular ring on the ground plane circuit (**g**) Inductive and capacitive effect between radiating and ground plane (**h**) Feedline circuit.

In the proposed antenna structure, a rectangular antenna with two corner notches, a microstrip line feed, and a rectangular ring slot on the ground plane is considered for circuit theory analysis. According to antenna theory, as presented in the literature^18–20^, an equivalent circuit of a two-port antenna with geometry shown in Fig. 2(a) is presented in Fig. 2(b). The equivalent circuit (cf. Figure 2(b)) consists of: a conventional rectangular patch antenna circuit (cf. Figure 2(c)), a notch equivalent circuit (cf. Figure 2(d)), a notch-loaded patch antenna circuit (cf. Figure 2(e)), the equivalent circuit of a rectangular ring slot on the ground plane (cf. Figure 2(f)), the circuit of inductive and capacitive effects between the radiating and ground planes (cf. Figure 2(g)), and a microstrip feedline circuit (cf. Figure 2(h)).

**Table 1:** Elemental value of lumped element circuits of Fig. 2.

Element	Value	Element	Value	Element	Value
${R}_{p}$	150 Ω	${C}_{n}$	0.8pF	${C}_{r}$	0.8 pF
${L}_{p}$	0.2 nH	${L}_{c}$	3.2 nH	${\Delta\:L}_{r}$	0.25 nH
${C}_{p}$	1.2 pF	${C}_{c}$	7.3 pF	${\Delta\:C}_{r}$	0.5 pF
${R}_{n}$	150 Ω	${R}_{r}$	250 Ω	${L}_{f}$	0.05 nH
${L}_{n}$	1.3 nH	${L}_{r}$	0.9 nH	${C}_{f}$	0.2 pF

Legends: ${R}_{p}$ - Patch resistor, ${L}_{p}$ – Patch inductor, ${C}_{p}$ -Patch capacitor, ${R}_{n}$ -Notch resistor, .

${L}_{n}$ -Notch inductor, ${C}_{n}$ -Notch capacitor, ${L}_{c}$ & ${C}_{c}$ – Coupling inductor and capacitor between patch and notch, ${R}_{r}$, ${L}_{r}\&{C}_{r}$ - Resistor, inductor and capacitor of rectangular ring on the ground plane, .

${\Delta\:L}_{r}\&{\Delta\:C}_{r}$ - Inductive and capacitive effect between radiating and ground planes, .

$${L}_{f}$$ & $${C}_{f}$$ – Feedline elements.

Finally, the equivalent impedance (${Z}_{in}$​) of the proposed circuit is calculated from Fig. 2(b), and thereafter, using the reflection coefficient Eq. (6) in terms of input impedance (${Z}_{in}$​) and characteristic impedance (${Z}_{0}$​), MATLAB code was written to obtain the circuit-theory reflection coefficient curve. After MATLAB simulation, the MATLAB-based circuit theory reflection coefficient curve (theoretical) is compared with the simulated result and is found to be in close agreement. The HFSS simulated and MATLAB simulated theoretical reflection coefficient curves are illustrated in Fig. 1. Theoretical and HFSS-simulated reflection coefficient results for the two-port antenna (Ref. design-4) are found to be in coherence, with only a very marginal difference (cf. Figure 1). Hence, the proposed equivalent circuit (cf. Figure 2(b)) of the two-port antenna validates well with the HFSS-simulated results. The elemental values of the circuit elements, as shown in Fig. 2, are also listed in Table 1.

6 $$\mathrm{Reflection\:coefficient\:(}\mathrm{d}\mathrm{B}\mathrm{)}=20\mathrm{log}\left|\frac{{Z}_{0}-{Z}_{in}}{{Z}_{0}+{Z}_{in}}\right|$$

### Parametric analysis of two port MIMO antenna

**Fig. 3:**
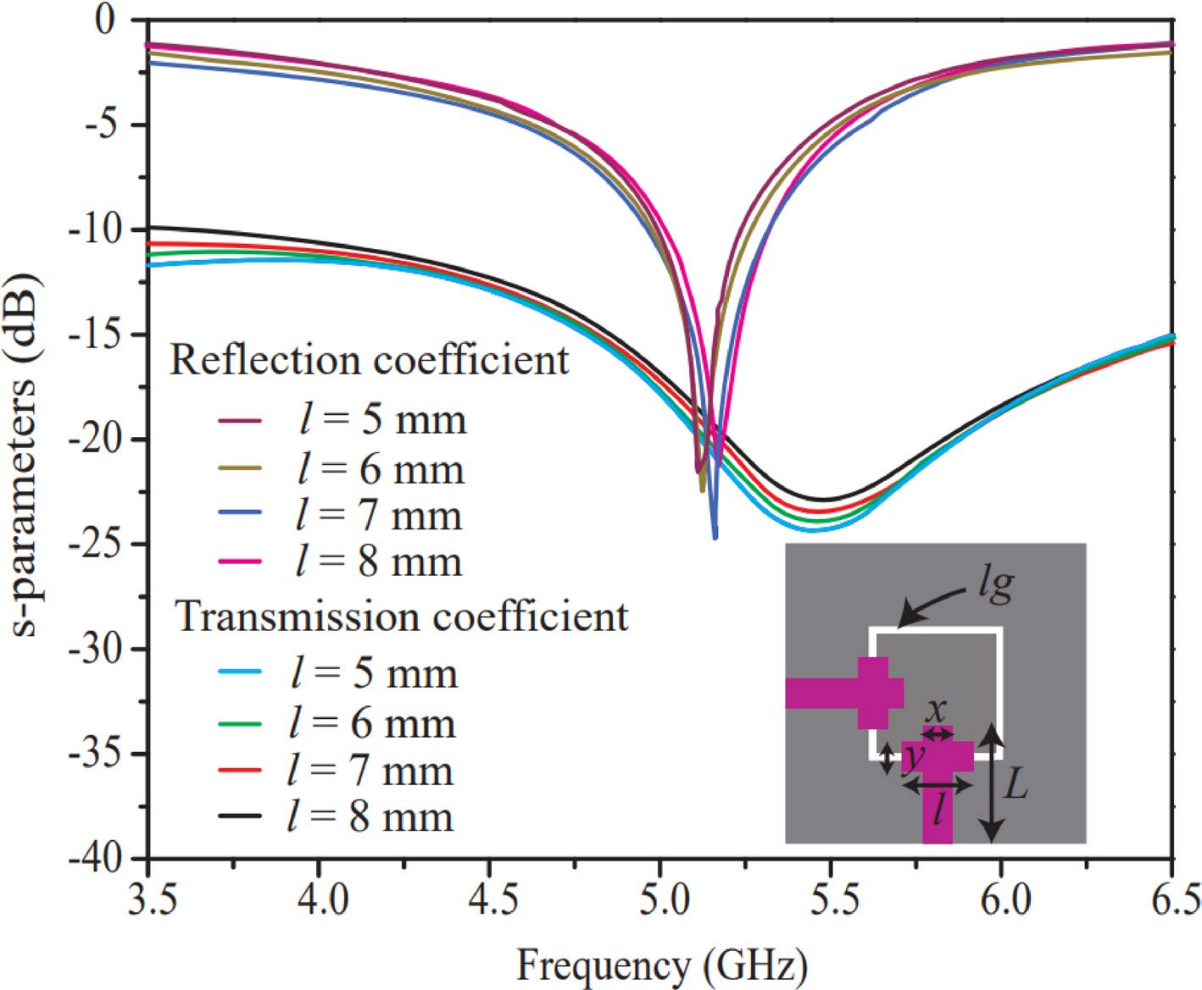
Effect of length (*l*) of small horizontal rectangular radiator.

**Fig. 4:**
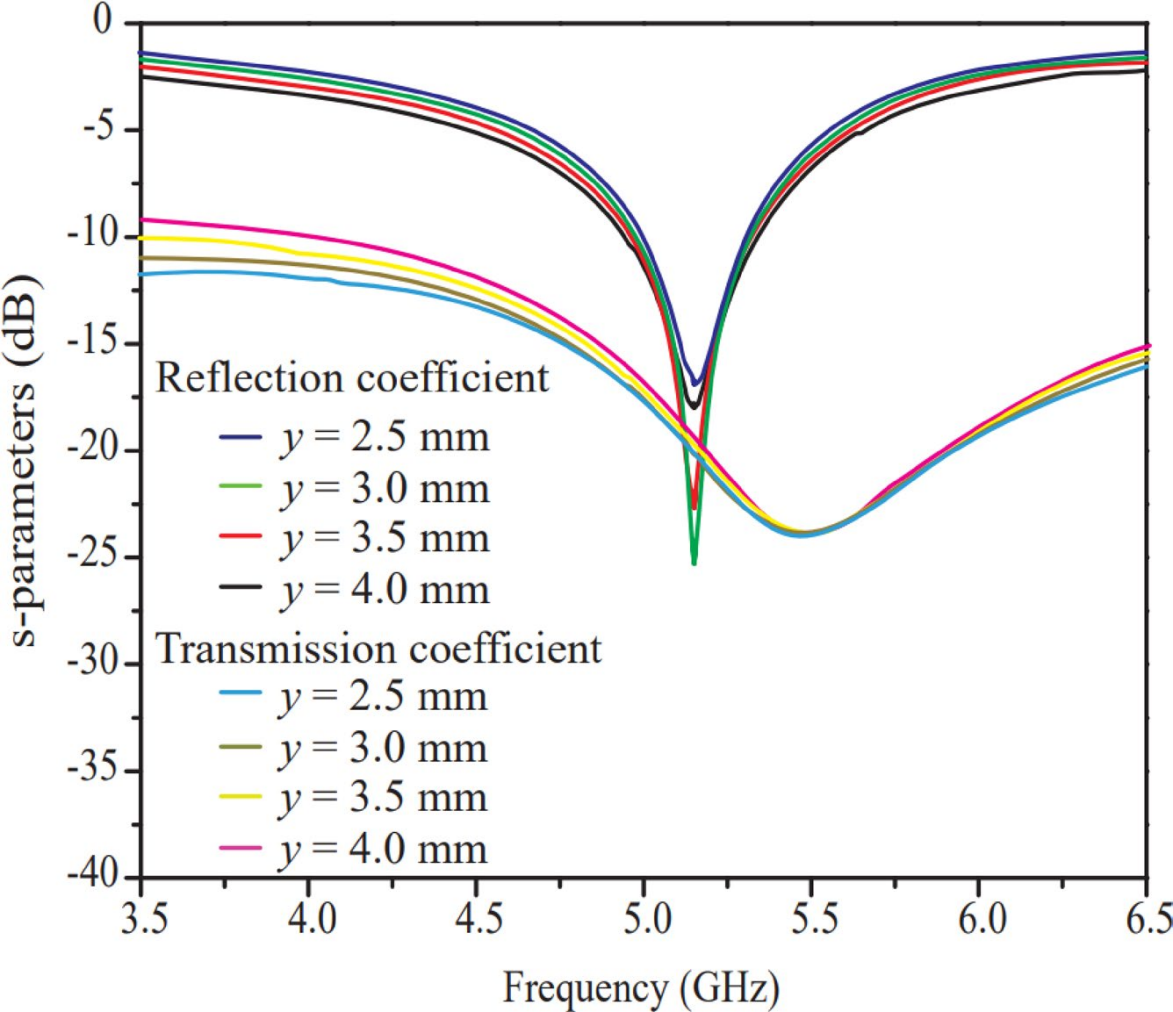
Effect of width (*y*) of small horizontal rectangular radiator.

**Fig. 5:**
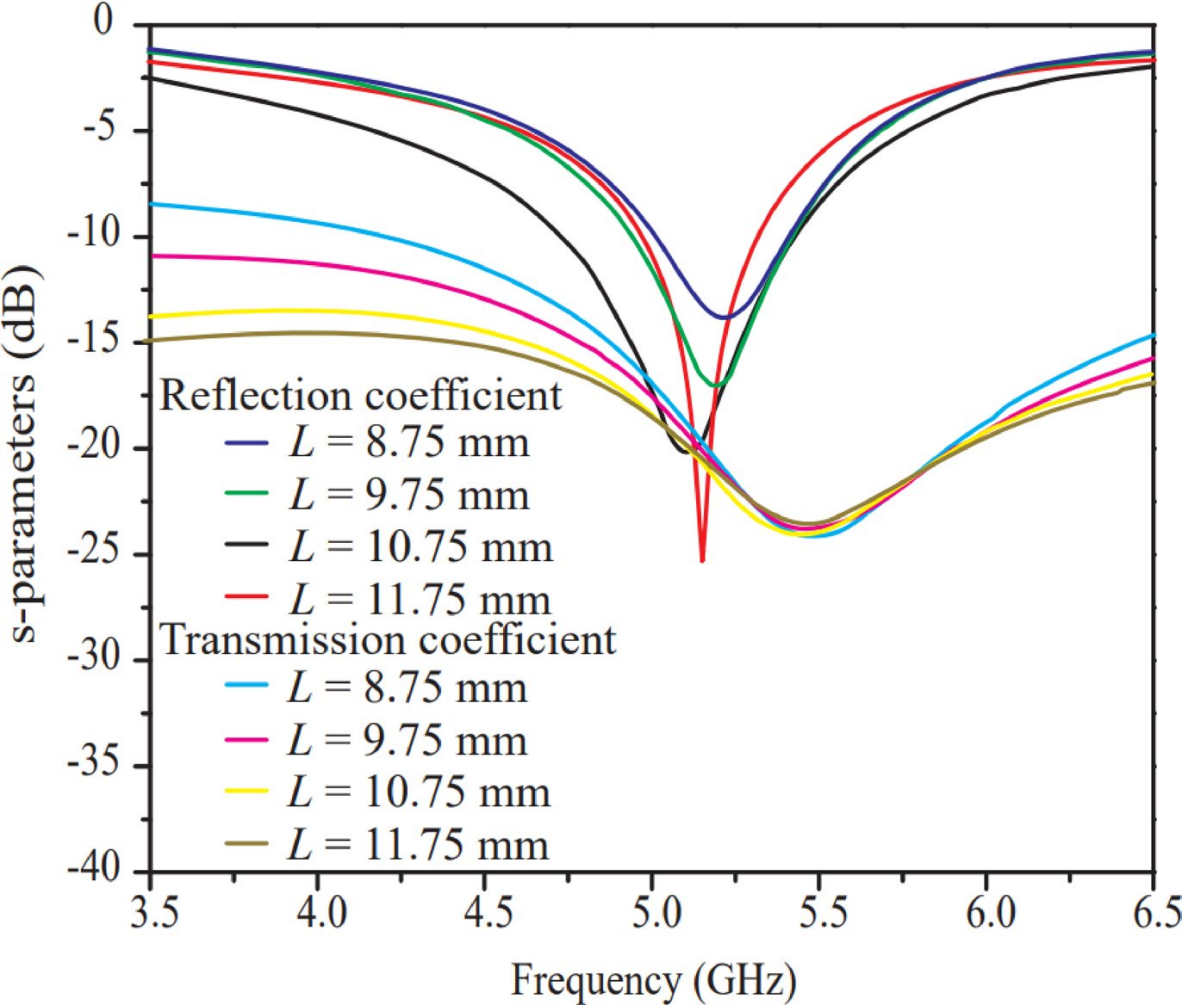
Effect of length (*L*) of long vertical rectangular radiator.

**Fig. 6:**
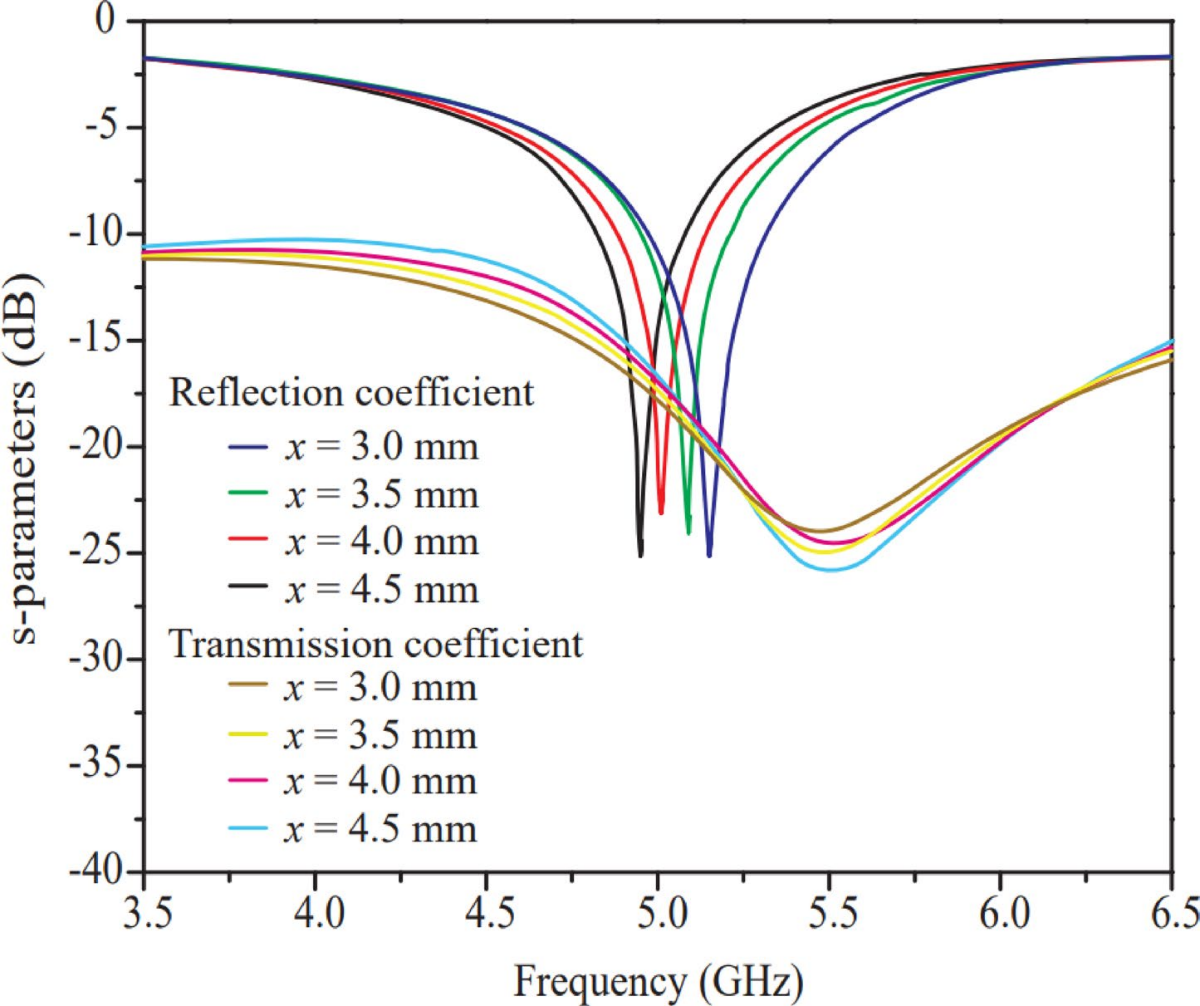
Effect of width (*x*) of long vertical rectangular radiator.

**Fig. 7:**
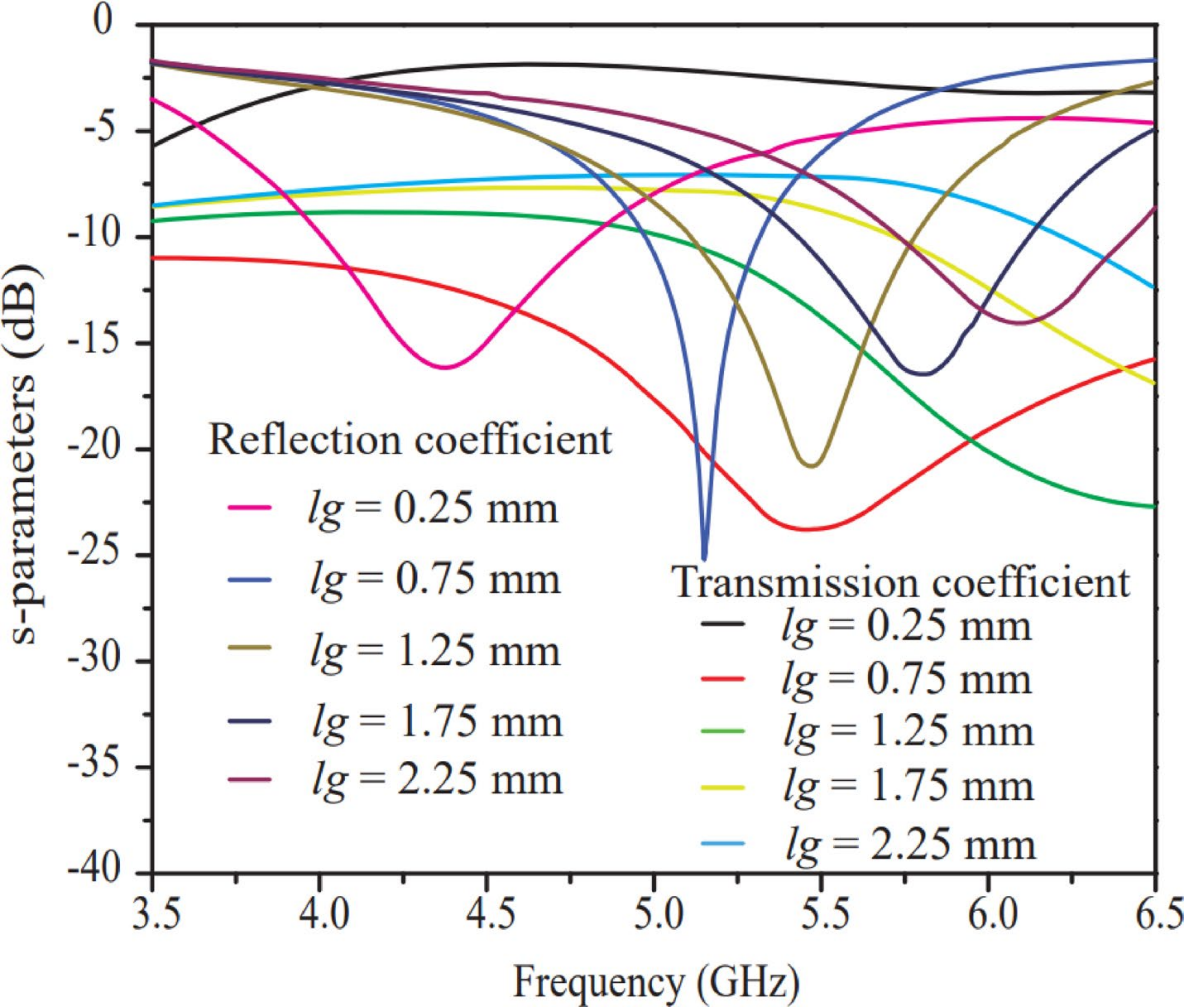
Effect of width (*lg*) of rectangular ground plane ring.

Before the implementation of the 8-element MIMO antenna, a parametric analysis of the proposed two-port antenna is carried out in this section. The parametric analysis, in terms of the length and width of the radiating elements (*l*,* y*,* L*,* x*) and the width of the ground-plane rectangular ring (${l}_{g}$​), is illustrated in Figures (3)–(7) and discussed accordingly. Figure 3 shows the effect of the horizontal length (${l}_{g}$), as indicated in the inset of Fig. 3. The length range is chosen between 5 and 8 mm with a step size of 1 mm, resulting in four iterations. Reflection and transmission coefficient curves are analyzed with variation in the length. From Fig. 3, it is observed that the antenna performs efficiently at a horizontal length of 7 mm. The antenna offers a good degree of reflection (−25 dB) and transmission (−22 dB) coefficients because this length provides perfect impedance matching. It is also noticed that the horizontal length does not produce good isolation beyond 7 mm length, because the distance between the two elements reduces significantly with further increments in horizontal length.

The vertical width (*y*) of the two-port antenna, varied between 2.5 and 4 mm with a step size of 0.5 mm (resulting in four iterations), is considered for parametric analysis. The effect of vertical width (*y*) variation in terms of reflection and transmission coefficient curves is shown in Fig. 4. The vertical width (*y*) does not affect the resonant frequency, but it changes the reflection coefficient value. It is observed that the antenna produces a good reflection coefficient curve (better than − 25 dB) at *y* = 3 mm compared to other iterations. On the other hand, the isolation of the antenna at the resonant frequency remains more or less the same, but this width significantly affects the reflection coefficient curve due to impedance matching. Hence, after studying all iteration results, it is concluded that a 3 mm width provides optimal performance.

The effect of the vertical length (*L*) of the two-port antenna is also analyzed with four iterations in the 8.75–11.75 mm length range with a 1 mm step size. The variation of reflection and transmission coefficients with respect to vertical length is shown in Fig. 5. A significant change in the reflection coefficient curve is observed in Fig. 5. This change is attributed to the vertical length (*L*), as it is an integral part of the microstrip feed line and the patch of the antenna. The antenna shows optimal results at an 11.75 mm vertical length among all four iterations. At 11.75 mm vertical length, a trade-off between the reflection and transmission coefficients is observed, and the antenna resonates at this length with low power loss and high isolation compared to other iterations.

The horizontal width (*x*) varies in the 3–4.5 mm range with a 0.5 mm step size, resulting in four iterations for parametric analysis. Reflection and transmission coefficient curves with respect to the horizontal width (*x*) are illustrated in Fig. 6. The resonant frequency changes with small variations in the horizontal width (*x*). As shown in Fig. 6, it is observed that the resonant frequency shifts toward higher frequencies when the horizontal width (*x*) decreases, and vice versa. The shifting of the resonant frequency with respect to the horizontal width (*x*) is expected because, according to antenna theory, the patch width and resonant frequency have an inversely proportional relationship. The proposed antenna shows good isolation and reflection coefficient behavior at a 3 mm horizontal width among all iterations. Isolation at 3 mm is better compared to other iterations because this width provides sufficient spacing between two adjacent elements, thereby reducing interference in the MIMO system. Hence, a 3 mm horizontal width (*x*) is chosen as the optimal dimension for the proposed antenna design.

The rectangular ring slot on the ground plane modifies the current distribution, which changes the inductive (${L}_{r}$) and capacitive (${C}_{r}$) effects of an equivalent circuit of the rectangular ring, leading to variations in resonant frequency, impedance matching, bandwidth, and surface waves of the antenna. The effect of the rectangular ring width (*l*g) on transmission and reflection coefficient performance is analyzed in Fig. 7. A range of 0.25–2.25 mm with a step size of 0.5 mm and a total of five iterations is considered for this analysis. It is clearly shown in Fig. 7 that the resonant frequency increases with each increment in rectangular ring width, and at *l*g = 0.75 mm, the antenna exhibits excellent reflection and transmission coefficient curves compared to other iterations. A rectangular ring slot with properly selected dimensions on the ground plane improves antenna performance by suppressing surface waves and enhancing impedance matching. Overall, the patch dimensions (*l*,* y*,* L*,* x*) and rectangular ring slot dimension (*l*g​) are optimized through parametric analysis, and the optimal value (*l =* 7 mm, *y =* 3 mm, *L =* 11.75 mm, *x =* 3 mm, ​ *l*g = 0.75 mm) is chosen for the design, fabrication, testing, and validation of the 8-port antenna, as well as for further discussion.

### Development of 8 × 8 MIMO antenna system

**Fig. 8:**
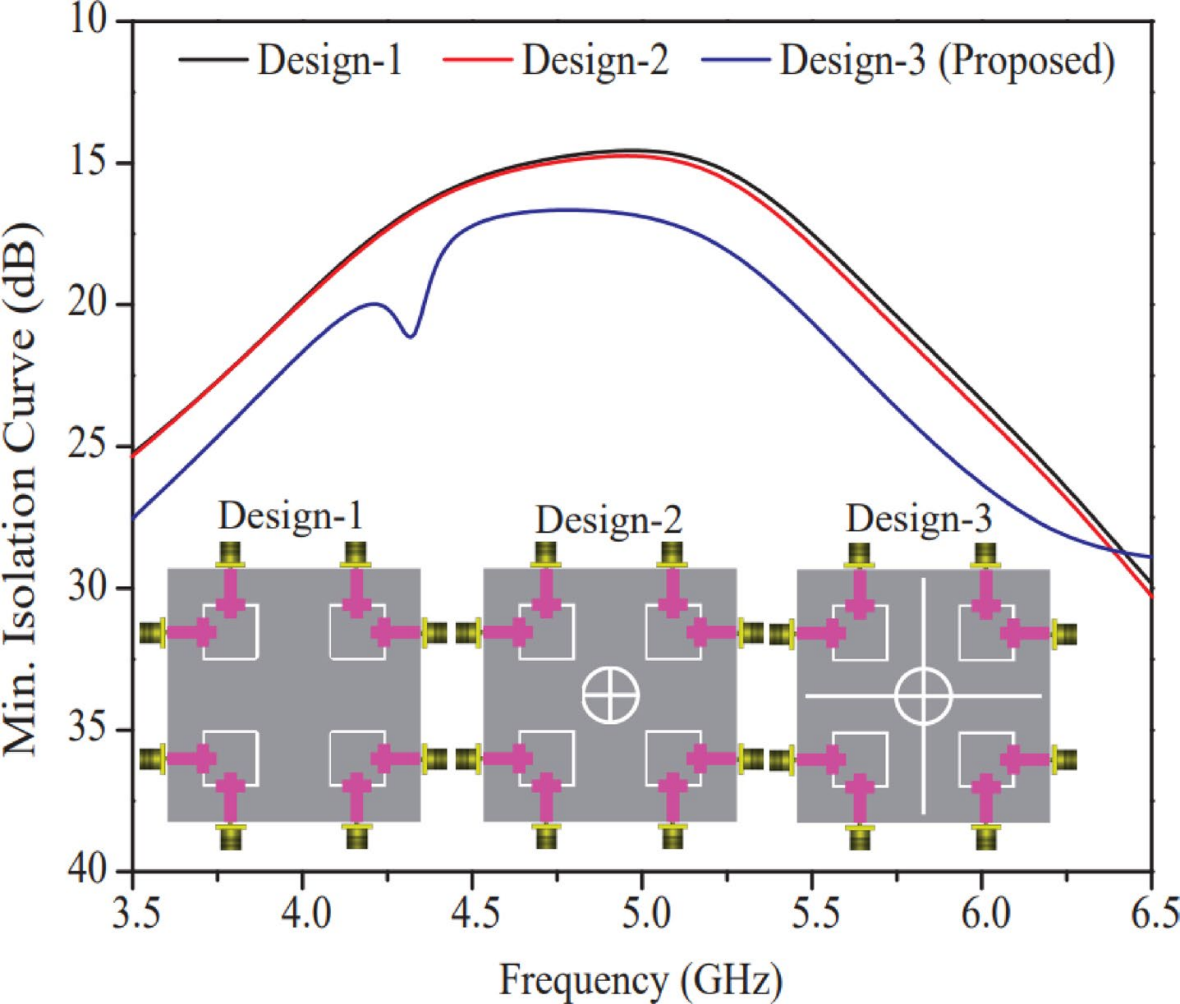
Min. isolation curves of different 8-port antenna designs (Design-1, Design-2, and Design-3 (proposed)).

After a systematic investigation, Ref. design-4 was chosen as the optimal design among all designs (Ref. design-1, Ref. design-2, Ref. design-3, and Ref. design-4) and three different step-wise 8-port MIMO antennas (Design-1, Design-2, and Design-3) were created, and their performance analysis in terms of isolation and reflection coefficient is presented in this section. Design-1 consists of a Ref. design-4 at the four corners of the RT/DUORID (5870 tm) substrate board (60 × 60 mm^2^). At the center of Design-1, an annular ring with a plus sign symbol geometry is etched out from the ground plane to create Design-2, whereas two additional rectangular rods (vertical and horizontal) are further etched out from the ground plane of Design-2 to create Design-3. Figure 8 shows the minimum isolation curves of three distinct eight-port antenna structures (Design-1, Design-2, Design-3). A close inspection of this representation reveals that Design-3 delivers a better isolation performance to Design-1 and Design-2 and has a minimum isolation of 17 dB at 5.2 GHz resonant frequency while designs-1 and Design-2 have a minimum isolation of 14 dB.

**Fig. 9:**
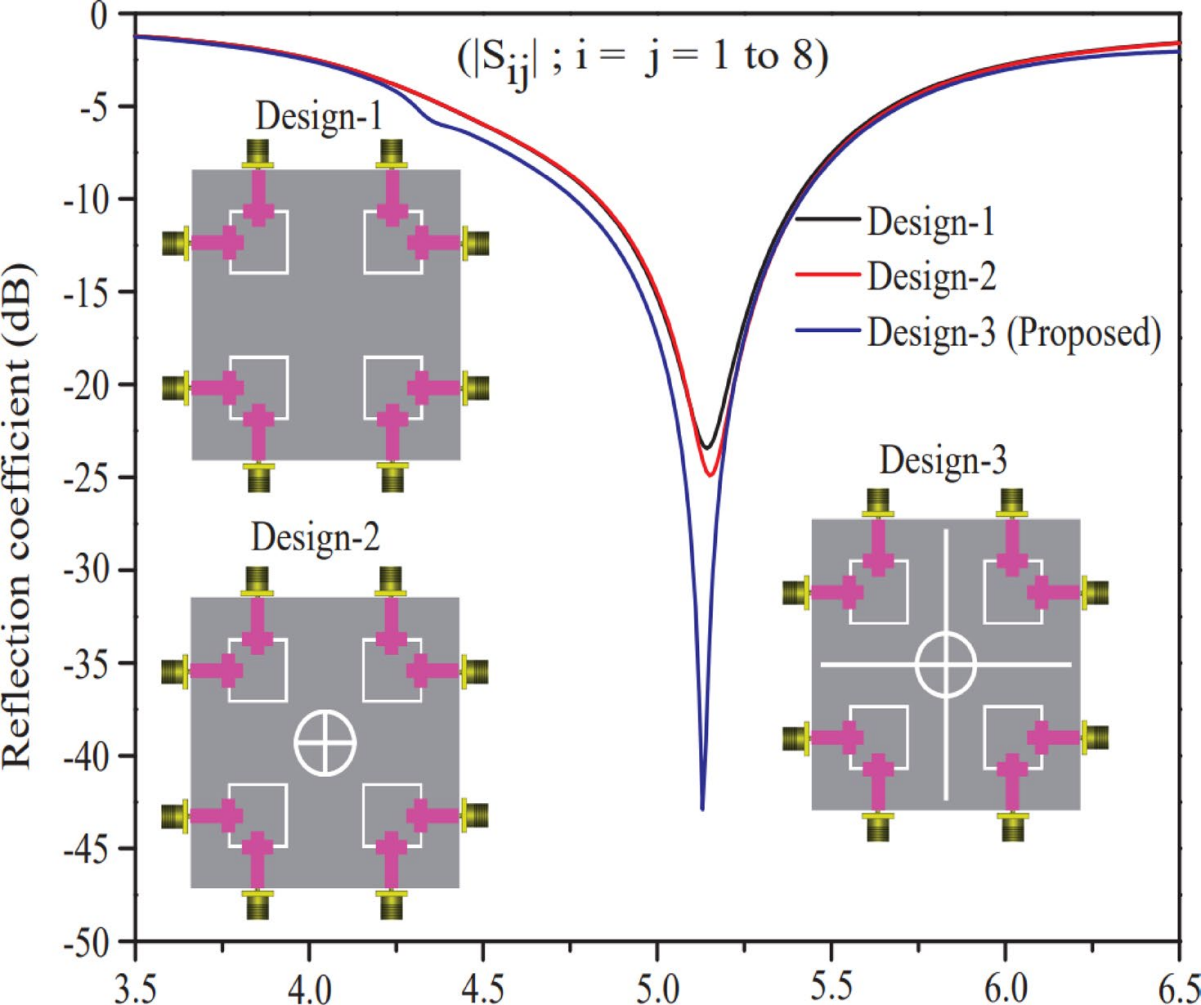
Reflection curves (|S_ij_|; i = j) of different 8-port antenna designs (Design-1, Design-2, & Design-3).

The reflection coefficient performance of the Designs (Design-1, Design-2, and Design-3) is portrayed in Fig. 9. A maximum bandwidth of 700 MHz (4.75–5.45 GHz) and minimum return loss (−43 dB reflection coefficient at 5.2 GHz) for Design-3 were obtained. Another hand Design-1 & Design-2 produce small bandwidth and large power loss as compared to Design-3. Therefore, based on the performance as shown in Figs. 8 and 9, Design-3 is selected as an outperform antenna structure among Design-1, Design-2 & Design-3.

## Effect of rectangular ring, annular ring and plus shaped slots on ground plane

Artificial ground structure (centered annular ring and plus shaped slot) as shown in Design-3 of Fig. 9, which serves as to enhance isolation between antenna elements, are formed when features such as rectangular rings^21^, annular rings^22^, and plus-symbol-shaped slots^23^ are etched away from the bottom plane of the MIMO antenna. By strategically altering the bottom plane surface current distribution, these etched patterns successfully prevent undesired coupling between nearby radiating elements. In the absence of such structures, MIMO performance is deteriorated by mutual coupling caused by surface waves and near-field interactions between antenna elements. The proposed structure reduces undesired electromagnetic interference between parts by adding more capacitance and inductance, changing the ground plane impedance characteristics, and producing a band-stop effect at specific frequencies (cf. Figures 8 and 9). Therefore, strategically etching rectangular rings, annular rings, and plus-symbol slots on the ground plane serves as an effective passive technique to minimize mutual coupling (up to −33 dB) and enhance the isolation characteristics of a MIMO antenna. These slots also function as electromagnetic bandgap (EBG) structures^24^, which prevent the propagation of surface waves that contribute to coupling. The modified current distribution forces the radiated energy to be more confined to each antenna element rather than leaking into adjacent elements, improving the isolation, which in turn leads to decreased envelope correlation coefficient (ECC), increased diversity gain, and improved overall MIMO system performance.

## Performance study of impedance matching and s-parameters

**Fig. 10:**
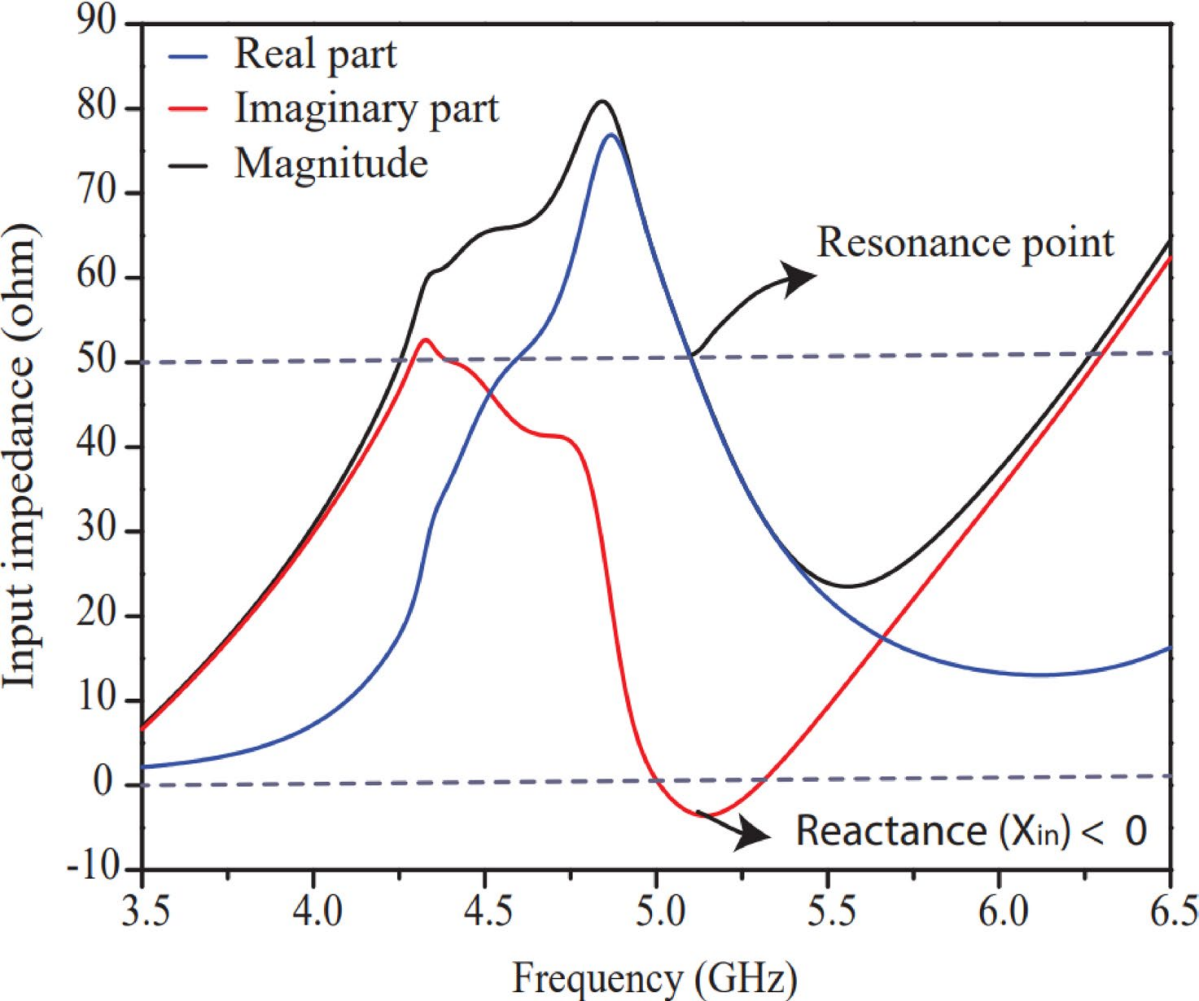
Input impedance curve for port-1 of Design-3.

This section discusses the performance of input impedance & s-parameter of the intended antenna. An equation-5 is used to select width (*x*) of feedline to match the antenna perfectly at 50 Ω characteristic impedances. Input impedance curve (real part, imaginary part and magnitude) of Design-3 for port-1 is shown in Fig. 10. Antenna seems perfectly matched at 5.2 GHz resonant point and shows 50 Ω input impedance. Reactive part of input impedance is around zero while real part and magnitude of input impedance approach 50 Ω impedance value. The performance of input impedance of Design-3 makes the antenna suitable for efficient transmission because of low power loss and perfectly match design.

**Fig. 11:**
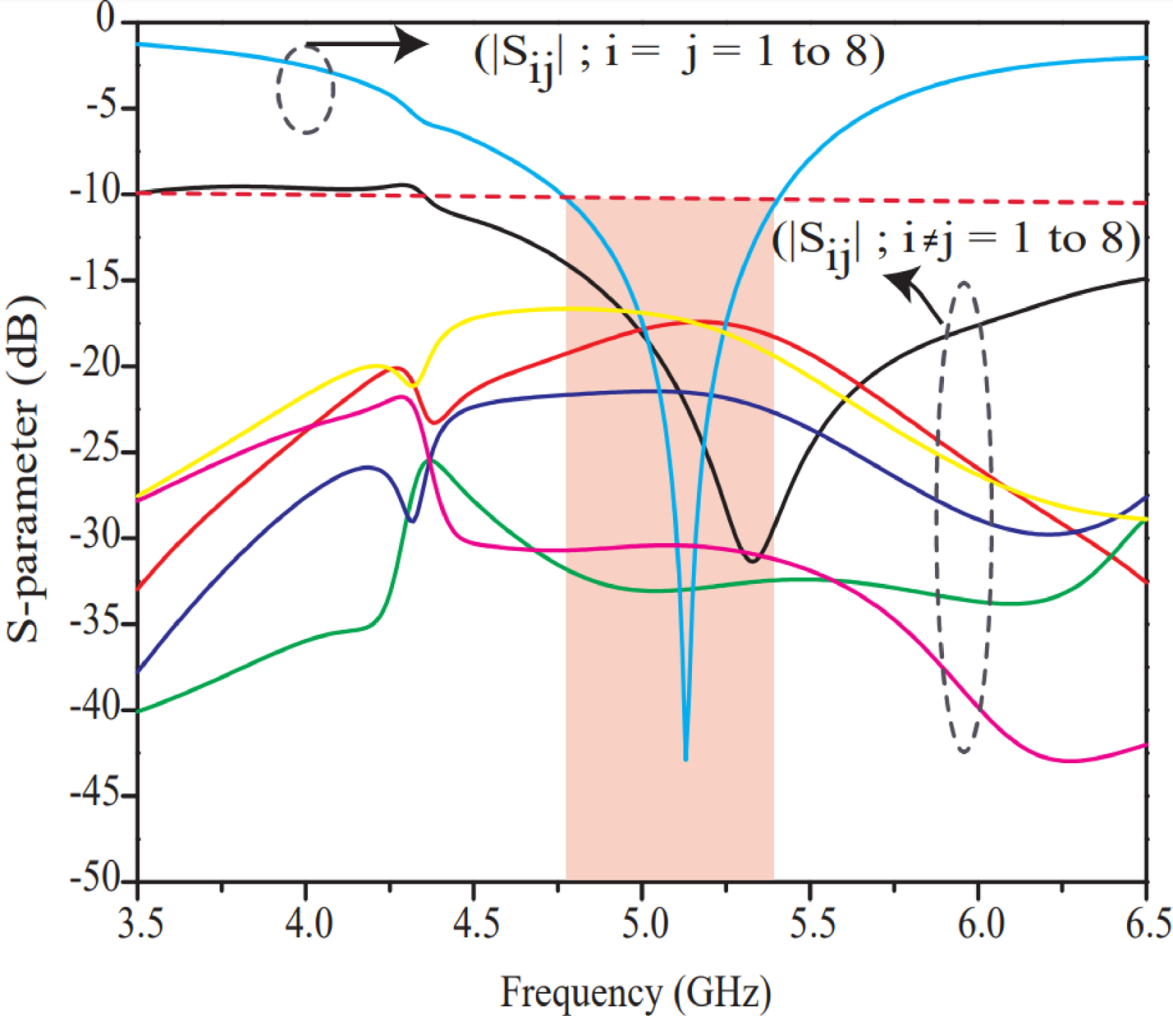
S-parameter performance of 8-port MIMO antenna Design-3 (proposed).

Figure 11 shows s-parameters of the proposed 8-port antenna wherein, it is clearly observed that isolation of the proposed antenna offers high isolation (17–33 dB), wideband bandwidth (700 MHz), perfect impedance matching (50 Ω) and low power loss (−43 dB reflection coefficient at resonate frequency). Owing to the above study it is concluded that the 8-port MIMO antenna (Design-3) outperforms as compared to Design-1 and Design-2 and is chosen as an optimal design for fabrication, testing, validation and further discussion. In Fig. 11, ${S}_{ij}$ represents reflection coefficient when $i=j,$ whereas if $i\ne\:j,$ ${S}_{ij}$ represents transmission coefficient.

## Antenna design configuration and fabrication

**Fig. 12:**
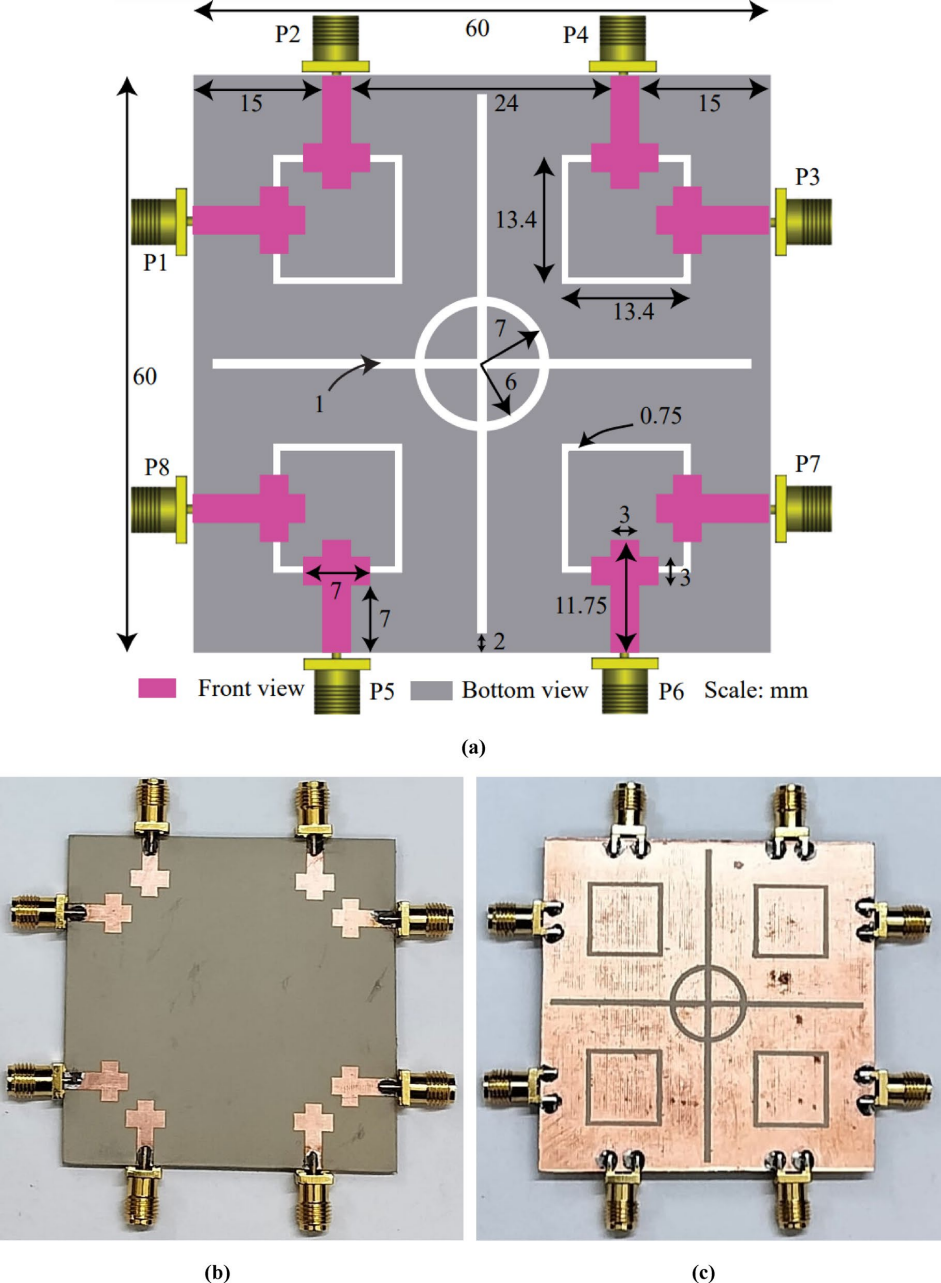
(a) Top and bottom view geometry of the proposed antenna (b) Front view photograph of the fabricated antenna (c) Bottom view photograph of the fabricated antenna.

The isolation, reflection coefficient, coupling coefficient, and input impedance of the suggested antenna (Design-3) were examined in the section before this one. The HFSS EM tool was used to assess its performance, and the simulated outcomes were verified by comparing them to measured data. The fabrication of the proposed antenna prototype was made using laser cutting and CNC machining from a DXF file of the antenna for testing and validation. The antenna geometry consists of two orthogonal elements of Ref. design-4 at four corners on top plane and rectangular ring at each corner and plus symbol sign along with centered annular ring are etched out from ground plane of the RT/DUORID (5870 tm) substrate board (60 × 60 mm^2^). Figure 12 (a) consists of a Neatly labeled front-and-bottom-view configuration of the suggested antenna. Fabrication and testing were conducted in antenna laboratory to validate the simulated and predicted results of the proposed configuration. The fabricated images of top and bottom views configuration are depicted by Figures. 12 (b) and (c), respectively.

## Antenna performance and results (simulated and experimental) validation

**Fig. 13:**
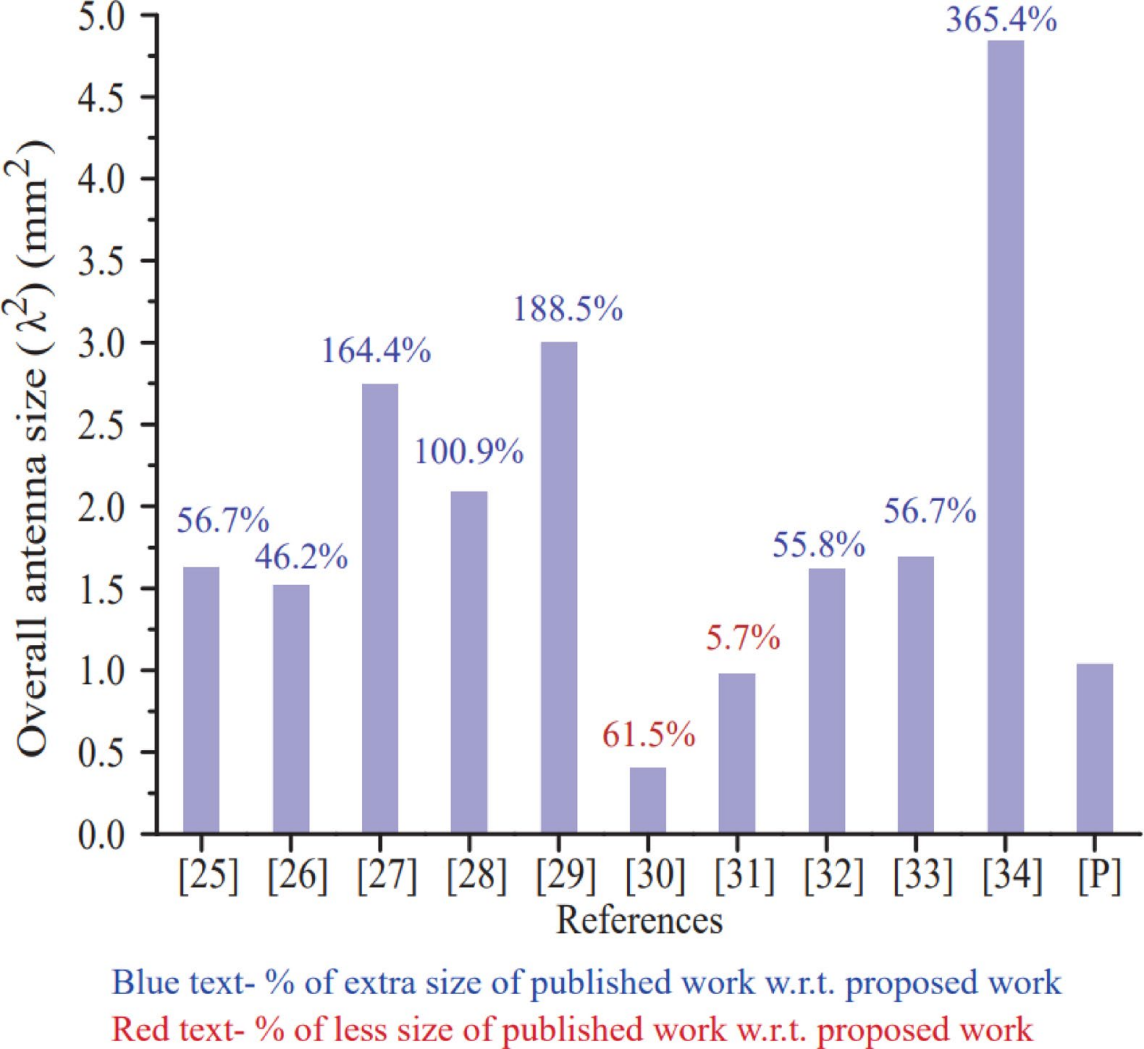
Comparison bar chart of overall antenna size.

The performance such as far-field parameters (gain and efficiency), s-parameters (reflection and transmission coefficient parameters), MIMO characteristics (envelop correlation coefficient (ECC), diversity gain (DG), mean effective gain (MEG), total active reflection coefficient (TARC) and channel capacity loss (CCL)) of the proposed antenna in this section is considered. In addition, the proposed antenna [P] is put into comparison with the 8-port antennas published in the few years back. Table 2 summarizes the results where the metrics i.e. antenna size, resonating frequency, bandwidth, efficiency, gain, isolation, ECC and CCL are mentioned for the proposed antenna as well as those available in references^25–34^. Based on second column of Table 2; Fig. 13 is used to compare the overall antenna size occupied by the antenna over the substrate board.

**Table 2:** Relative performance analysis of eight-port antennas.

[Ref.] Year	Overall size (mm^2^)	${\boldsymbol{f}}_{\boldsymbol{r}}$ (GHz)	−10dB BW (GHz)	${\boldsymbol{\eta}}_{\mathrm{rad}}$ (%)	Max. Gain (dBi)	Peak Isolation (dB)	ECC (abs)	CCL (Bit/Sec/Hz)
^25^ 2024	1.75λ × 0.93λ	3.5	3.3–3.8	89.8	NR	11	0.03	0.02
^26^ 2024	1.75λ × 0.87λ	3.5	3–3.9.9	60	5.3	10	0.19	0.4
^27^ 2024	2.35λ × 1.17λ	4.7	4.38–5.19	90.57	3.4	13.9	0.066	NR
^28^ 2024	2.05λ × 1.02λ	3.5/4.1	3.3–4.2	70	4.25	14.5	0.02	0.4
^29^ 2024	2.16λ × 1.39λ	5.03	4.5–5.5	90	4	24	0.07	NR
^30^ 2024	0.63λ × 0.63λ	3.5	3.06–3.71	99.1	−1.98	40	0.00003	NR
^31^ 2024	0.99λ × 0.99λ	5.5	5.44–5.61	85	5.75	22	0.15	0.2
^32^ 2022	1.8λ × 0.9λ	3.6	3.4–3.8	70	3.5	17	0.03	NR
^33^ 2022	1.75λ × 0.93λ	3.5	3.4–3.6	75	6.5	17	0.065	NR
^34^ 2023	2.2λ × 2.2λ	6.08	6.01–6.14	90	4.72	21.17	0.0027	NR
[P] NA	1.02λ × 1.02λ	5.13	4.75–5.45	92.5	4.7	33	4.6×${10}^{-6}$	0.04

Legends: NR–Not reported, [P] –Proposed work, NA-Not applicable, ${f}_{r}$ – Resonating frequency, λ–free space wavelength, ${\eta}_{\mathrm{rad}}$ – Radiation efficiency, BW-Bandwidth.

From perusal of Fig. 13, it is observed that the proposed antenna is compact as compared to antennas^25–29,32–34^ and large as compared to antennas^30,31^. The proposed antenna reduces 56.7% antenna size with reference to antenna^25^, 46.2% antenna size with reference to antenna^26^, 164.4% antenna size with reference to antenna^27^, 100.9% antenna size with reference to antenna^28^, 188.5% antenna size with reference to antenna^29^, 55.8% antenna size with reference to antenna^32^, 56.7% antenna size with reference to antenna^33^ and 365.4% antenna size with reference to antenna^34^. However, antenna size of the proposed antenna is 61.5% and 5.7% large with respect to antennas^30^&^31^, respectively. The size of antennas^30^&^31^ is less compared to the proposed antenna but other performance parameters of antennas^30^&^31^ such as bandwidth and gain are comparatively less.

**Fig. 14:**
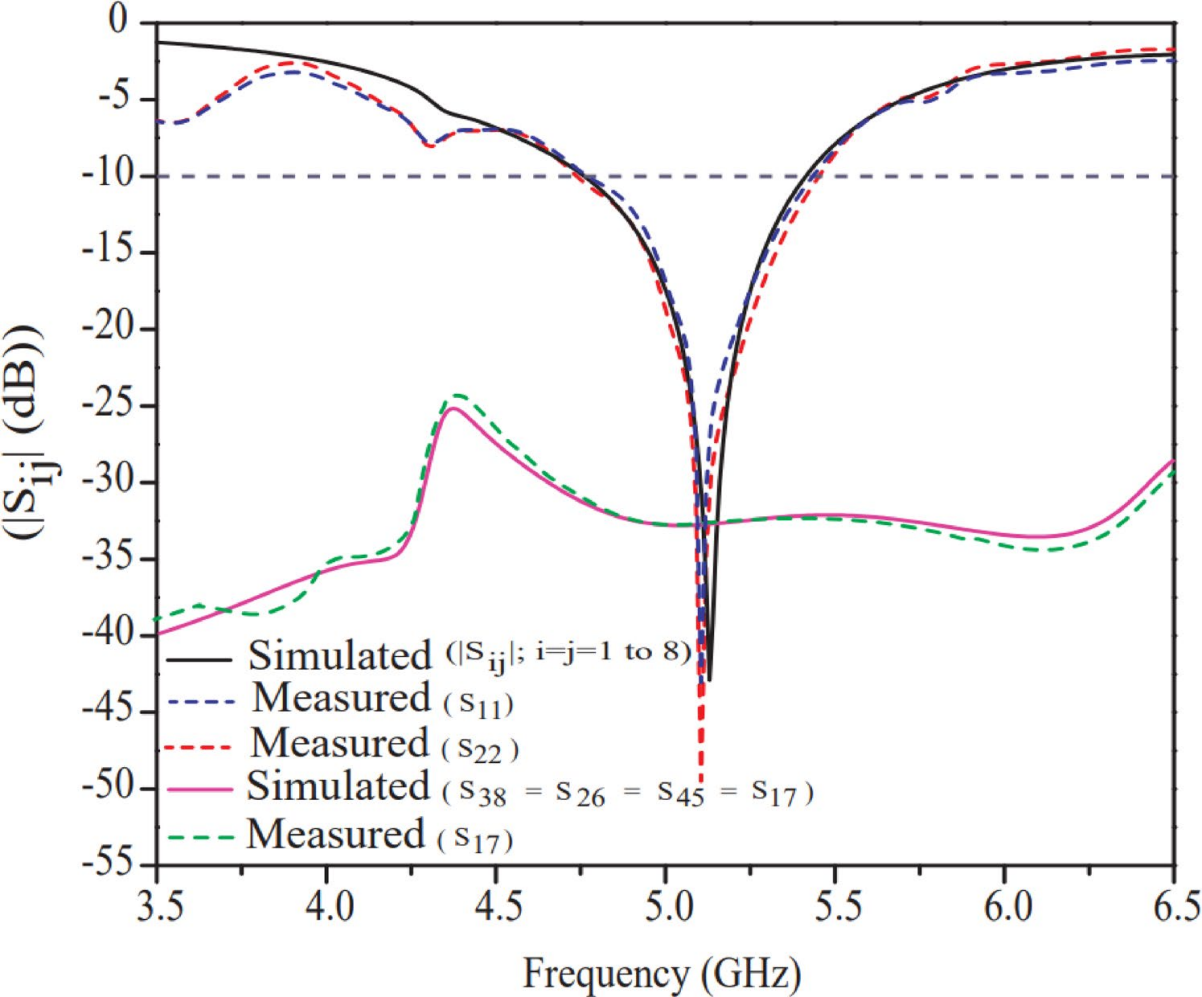
Simulated and measured reflection and transmission coefficient curve of the proposed antenna.

**Fig. 15:**
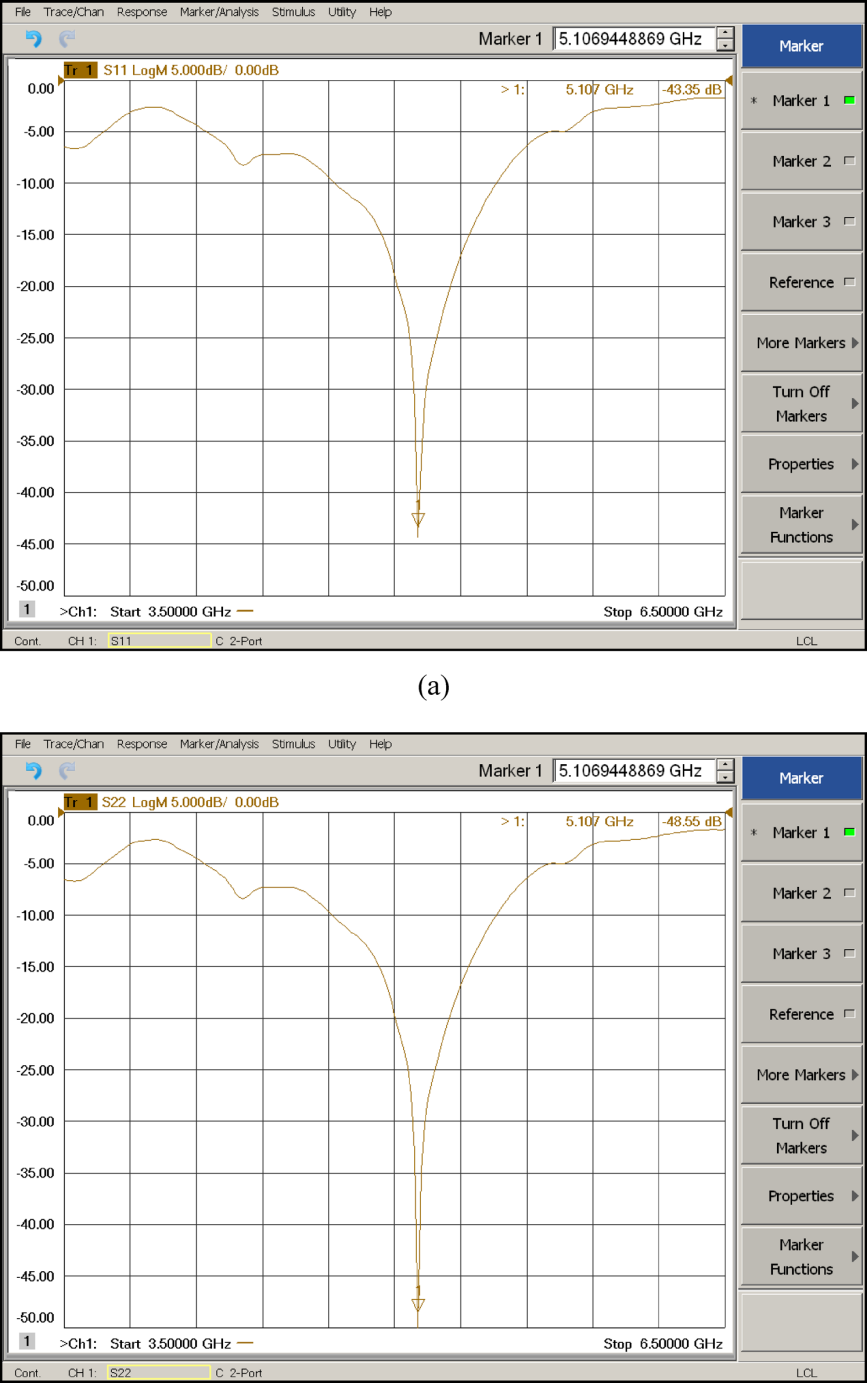
Experimental curves measured through VNA (N5274A) (a) ${|S}_{11}|$ (b) ${|S}_{22}|$.

The plot of simulated and measured reflection coefficient and transmission coefficient of the proposed antenna is shown in Fig. 14. The experimental curves of ${|S}_{11}|$ and ${|S}_{22}|$ measured through VNA (Vector Network Analyzer (N5274A)) are portrayed in Figs. 15 (a) & (b), which are well aligned with the simulated results. The measured results may vary for different ports of the MIMO antenna due to non-ideal conditions such as fabrication tolerances, soldering imperfections, slight asymmetries in antenna elements, conductor loss, dielectric loss, mismatch loss, environmental factors, and improper instrument calibration. For the proposed antenna, the reflection coefficients for port-1 ${|S}_{11}|$ and port-2 ${|S}_{22}|$ were tested, and only a very marginal difference was found between their results. On the other hand, the transmission coefficient between port-1 and port-7 was tested and validated with simulated results, as shown in Fig. 14. It has been noticed that the proposed antenna offers a wide bandwidth of 740 MHz (4.72–5.46) (simulated) and 680 MHz (4.75–5.43) (measured). The reflection coefficient plot for all 8-ports |(${S}_{i,j(i=j)}$)| is superimposed to each other and a minimum power loss (−50 dB reflection coefficient) is obtained at resonant frequency (5.2 GHz). The simulated curve is well validated with experimental curve as clearly depicted in Fig. 14.

The bandwidth (740 MHz) of the proposed antenna is large as compared to antennas 500 MHz^25^, 650 MHz^30^, 170 MHz^31^, 400 MHz^32^, 200 MHz^33^ and 130 MHz^34^ as reported in Table 2. However, the bandwidth of the proposed antenna is less than the antennas 900 MHz^26^, 810 MHz^27^, 900 MHz^28^ and 1000 MHz^29^ reported in Table 2. However, antennas^26–29^ have obtained comparatively large bandwidth with respect to the suggested antenna with scarification of other performance matrices such as antenna size, gain and diversity parameters.

Transmission coefficient curve with high isolation (33 dB) which is obtained through ports of P38, P26, P45, P17 is only shown in Fig. 14 and rest are omitted to avoid clustering in Fig. 14. A maximum isolation of 33 dB of the suggested antenna is obtained because of the unique isolator structure (centered annular ring and plus shaped slot) on the bottom plane.

The isolation of the intended antenna is significantly high with respect to the isolation of antennas 11dB^25^, 10dB^26^, 13.9dB^27^, 14.5dB^28^, 24dB^29^, 22dB^31^, 17dB^32^, 17dB^33^ and 21.17dB^34^ as indicated in Table 2. However, the isolation (40dB) of antenna^30^ is high as compared to the proposed antenna but it suffers from very low gain (−1.98dB). The transmission and reflection coefficient of the proposed antenna as presented in preceding sections are clearly conferred that the proposed antenna covers NR-n46 and n79 band for 5G applications with high isolation, mutual coupling and perfect matching.

**Fig. 16:**
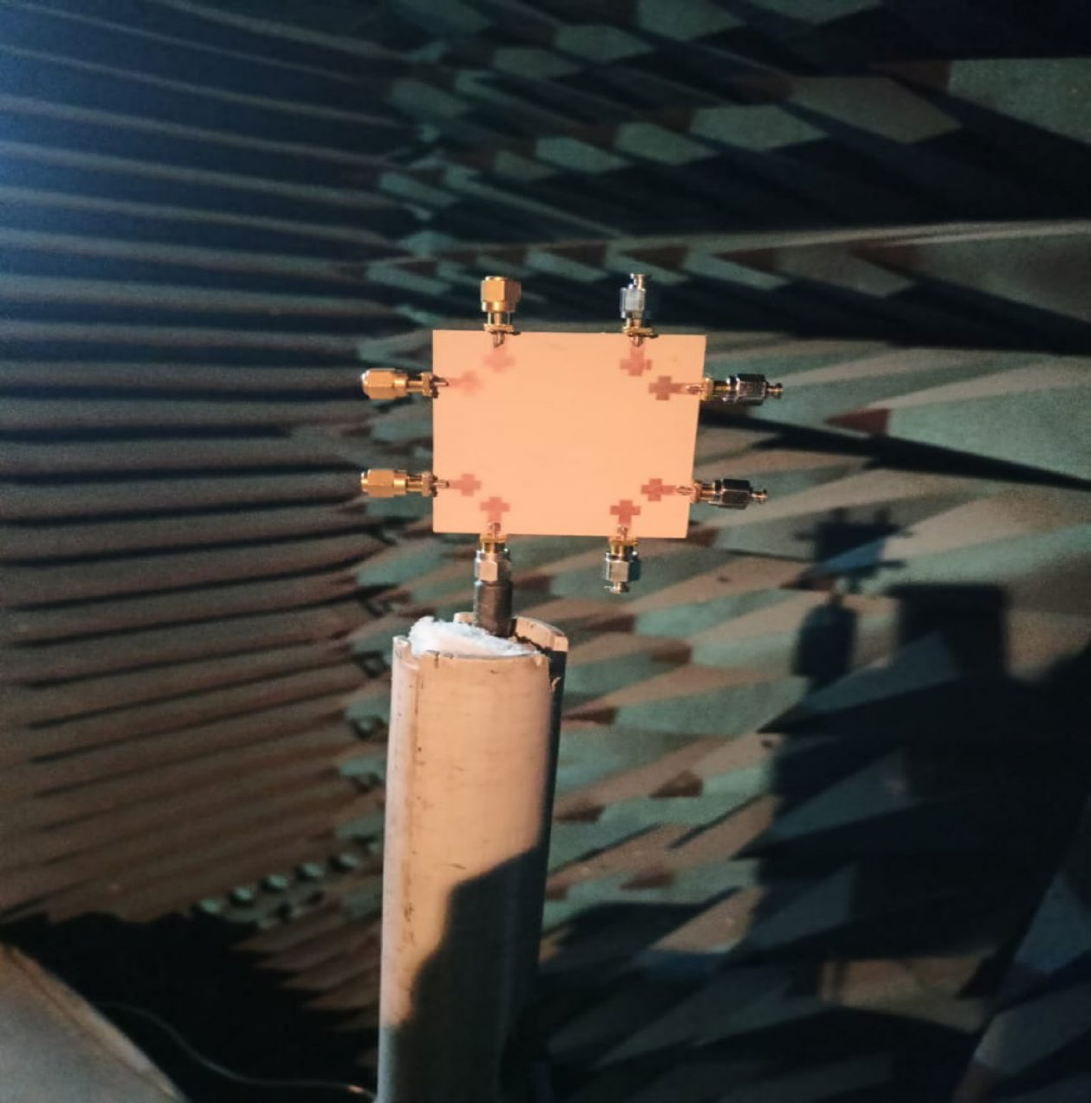
Measurement setup of the proposed antenna in an anechoic chamber with terminating loads.

**Fig. 17:**
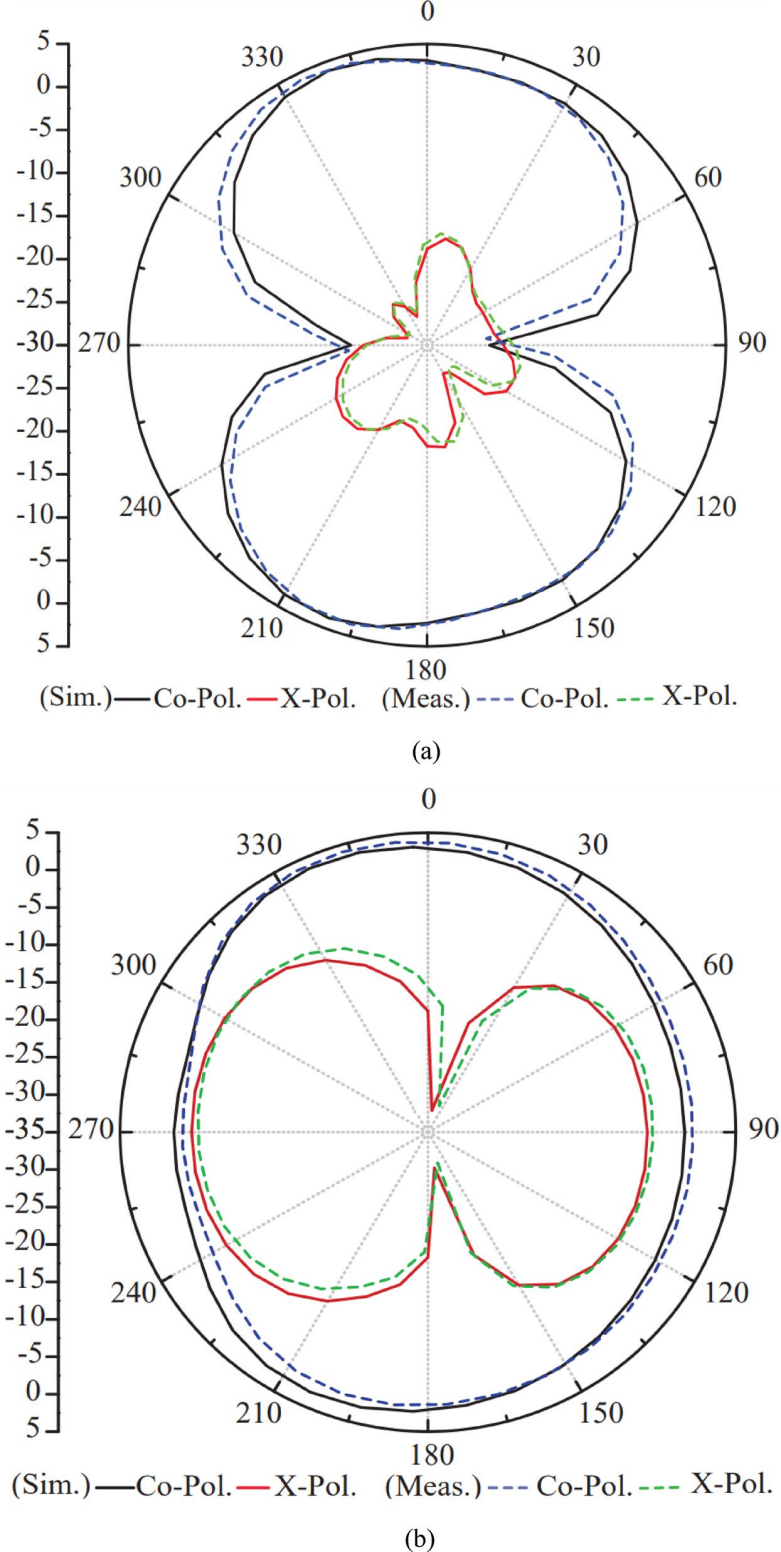
Co-Pol. & X-Pol. of the suggested antenna for port-1 (P1) at 5.2 GHz in **(a)** Electric plane **(b)** Magnetic plane.

The radiation parameters experiment of the suggested antenna is performed in an anechoic chamber measurement setup with rotating and controlling mechanism with reference antenna (horn antenna) as setup shown Fig. 16. During measurement unused ports are terminated with perfectly matched 50 ohm load to ensure accurate and reliable results. Using far-field data, sim. and meas. Co-Pol. & X-Pol. radiation patterns of the suggested antenna in electric and magnetic planes at 5.2 GHz are illustrated in Figures. 17 (a) & (b), respectively. From Fig. 17 (a), a directional gain distribution of the proposed antenna with numerical digit “8” shape structure is achieved which indicates efficient transmission in intended direction.

In other hand, low level cross polarization is also observed in Fig. 17 which has suppressed interference between orthogonally polarized waves. The magnetic plane (H-plane) radiation plot of the suggested antenna is depicted in Fig. 17 (b). It is seen that the intended antenna radiation characteristic in perpendicular to the antenna axis is omnidirectional and radiates equally in horizontal planes. The X-Pol. component level is substantially low with respect to co polarization. This characteristic is critical for beamforming and multiple-input multiple-output (MIMO) applications, where spatial diversity and signal quality are improved by reducing polarization leakage.

**Fig. 18:**
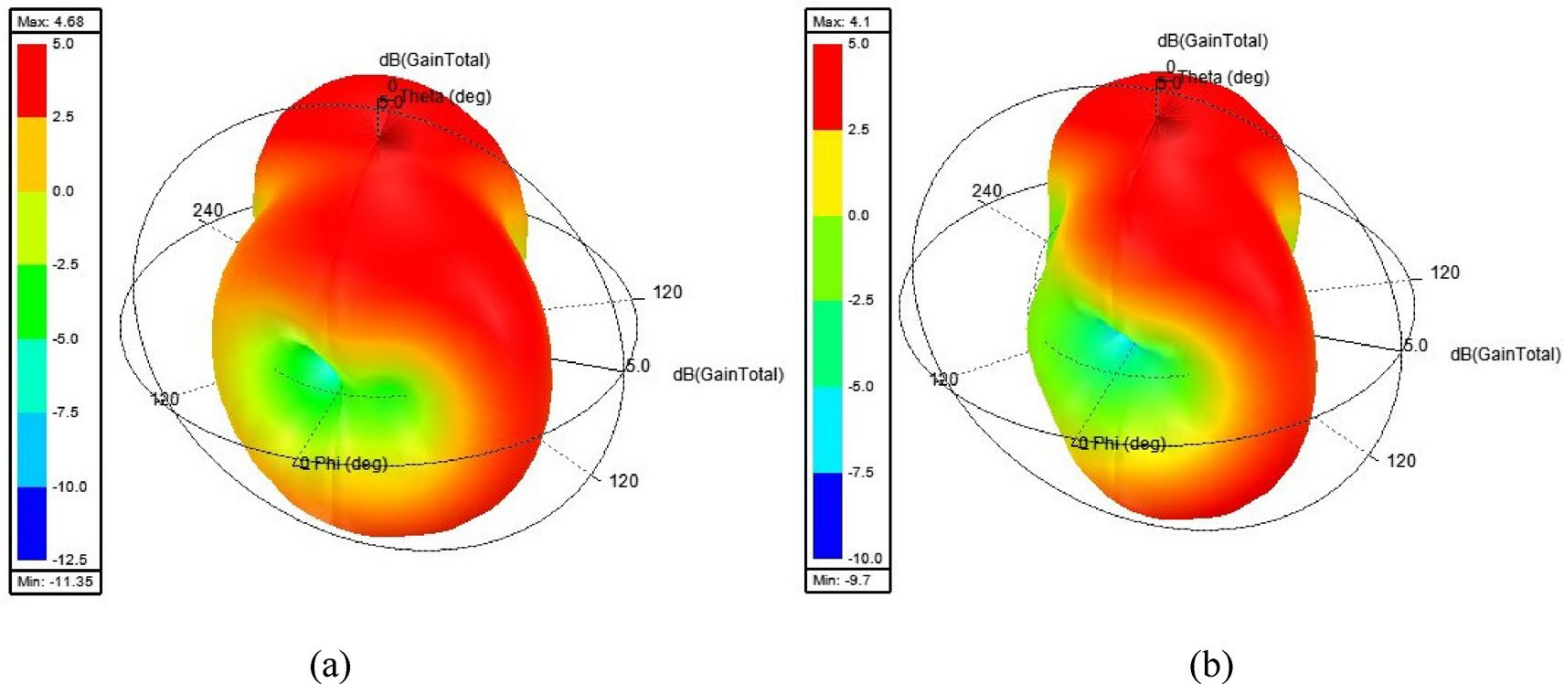
Three-dimensional antenna gain for port-1 (P1) at (a) 4.8 GHz (b) 5.2 GHz.

**Fig. 19:**
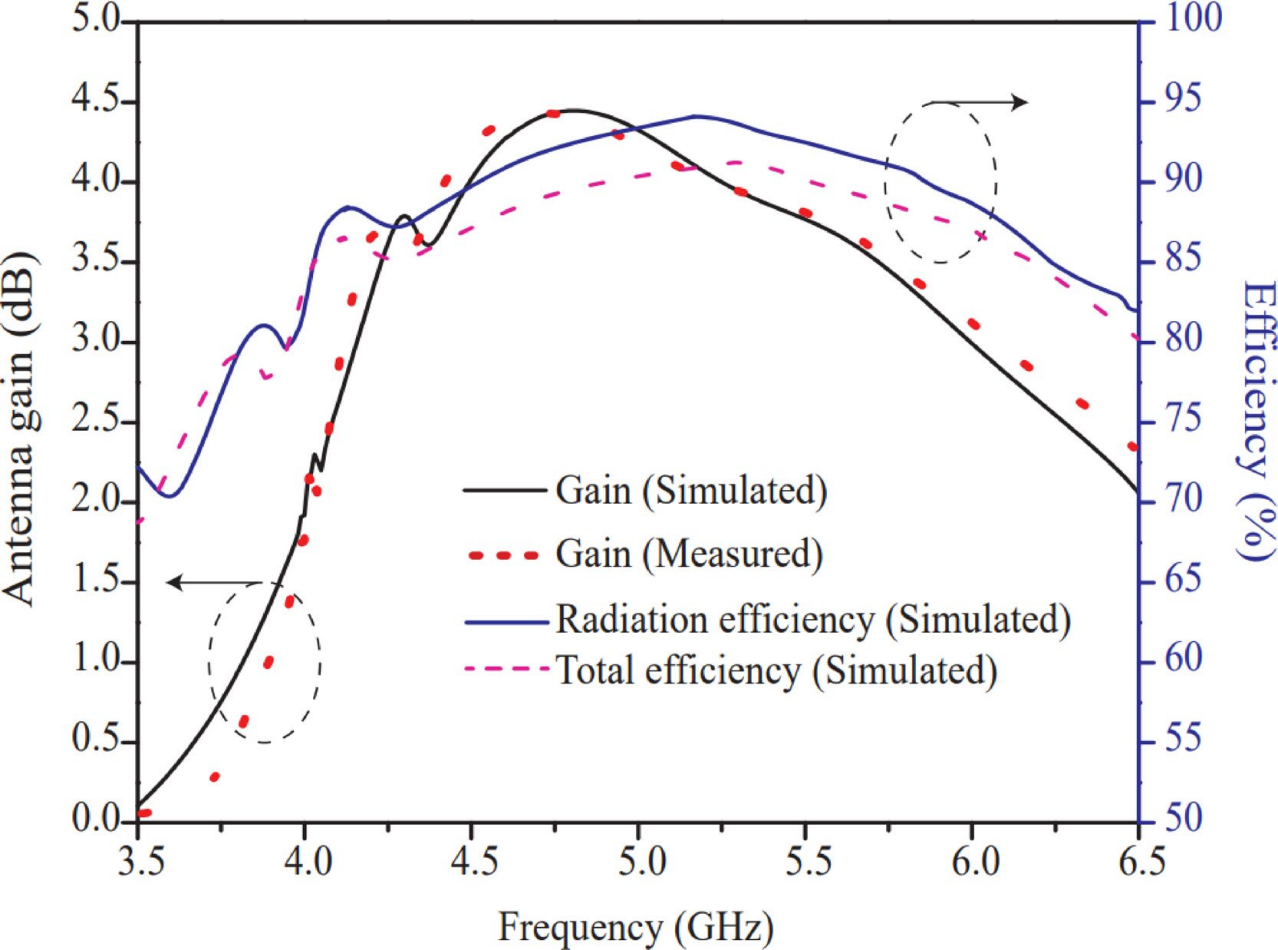
2D antenna gain and radiation efficiency curves for port-1 (P1).

The three-dimensional gain of the suggested antenna at two frequencies i.e. 4.8 GHz and 5.2 GHz are displayed in Figs. 18 (a) & (b), respectively. A peak antenna gain of the intended antenna of 4.68 dB at 4.8 GHz and 4.1 dB at 5.2 GHz is achieved. This gain has been achieved due to proper feeding technique, perfect impedance matching, optimized 8-port antenna structure and proper selection of substrate material. The gain of the suggested antenna is relatively high with respect to the antennas^27–30,32^; 3.4dB^27^, 4.2dB^28^, 4dB^29^, −1.98dB^30^ and 3.5dB^32^ and low with respect to the antennas^26,31,33^; 5.3dB^26^, 5.75dB^31^ and 6.5dB^33^ and equal to the antenna 4.7dB^34^ as indicated in Table 2. The antennas^26,31,33^ have obtained little bit high gain with compromise of other performance characteristics such as compactness, operating bandwidth, efficiency and diversity parameters. Moreover, the two-dimensional curve of the intended antenna with sim. and meas. results are portrayed in Fig. 19. The meas. gain of the antenna is calculated according to work reported in^35^ using Friis equation as given Eq. (7):

7 $${P}_{r}={\left(\frac{{\uplambda}}{4\pi{R}_{F}}\right)}^{2}{G}_{r}{G}_{t}{P}_{t}$$

where, ${R}_{F}$ – distance between transmitter and receiver, ${\uplambda}$ = guide wavelength, ${P}_{r}$- received power, ${G}_{t},{G}_{r}$ – transmitting and receiving antenna gain, ${P}_{t}$ – transmitted power.

From Fig. 19, it is stated that the gain is consistent in the entire resonating band and in concurrence with 3D gain as shown in Fig. 18. The simulated curves of radiation (${\eta}_{\mathrm{rad}}$) and total efficiency (${\eta}_{\mathrm{total}}$) are illustrated in Fig. 19, and the corresponding values are indicated on the right-side vertical axis. The measured curve of efficiency of the proposed antenna is omitted due to its complex measurement procedure. It is noticed that the radiation efficiency curve follows the gain curve and reached a maximum of 92.5% at 5.2 GHz. The efficiency (92.5%) of the suggested antenna is substantially large with respect to the antennas 89.8%^25^, 60%^26^, 90.57%^27^, 70%^28^, 90%^29^, 85%^31^, 70%^32^, 75%^33^ and 90%^34^ and lower than the antenna 99.1%^30^ as reported in Table 2. The antenna^30^ offers very high efficiency, but gain is negative which limits the application of the antenna.

Moreover, the total efficiency of the proposed MIMO antenna is calculated according to work^36^ using Eqs. (8) and (9), and is presented in Fig. 19. The total efficiency of the MIMO antenna represents effective efficiency, where all losses such as conductor loss, dielectric loss, and mismatch loss are considered, whereas only conductor and dielectric losses are considered for radiation efficiency. In conclusion, radiation efficiency relates the power accepted by the antenna to the radiated power, whereas total efficiency indicates how much of the source power is radiated, including losses due to mismatch and coupling. Hence, a lower value of total efficiency compared to the radiation efficiency of the MIMO system is expected. This fact can also be observed from the relationship between radiation efficiency and total efficiency, as mentioned in Eq. (8). From Fig. 19, it is observed that the total efficiency (90% at the resonant frequency) follows the radiation efficiency curve and is slightly lower due to the inclusion of mismatch loss. Figure 19 confirms that the proposed MIMO antenna transmits and receives signals efficiently with minimal loss.

8 $${{\upeta}}_{\mathrm{total}}={{\upeta}}_{\mathrm{rad}}\cdot\left(1-{\mathrm{TARC}}^{2}\right)$$

9 $$\mathrm{TARC}=\frac{\sqrt{\left|{\sum}_{i=1}^{8}{S}_{i1}+{\sum}_{m=2}^{8}{S}_{im}{e}^{j{{\uptheta}}_{m-1}}\right|}}{\sqrt{8}}$$

Where, TARC-Total Active Reflection Coefficient, ${S}_{i1}$- power delivered from port 1 to port *i*, ${S}_{im}$ - power delivered from port 1 to port *m*, ${{\uptheta}}_{m-1}$ – phase shift at (m-1)^th^ port.

Multiple input and multiple output (MIMO) antennas have been eminent in maximizing the channel capacity and in enabling the spatial multiplexing. The eight port MIMO diversity characteristics like ECC, DG, TARC, MEG and CCL have included in this section for antenna performance study. Separate discussion for each performance parameter is presented in forgoing paragraphs.

**Fig. 20:**
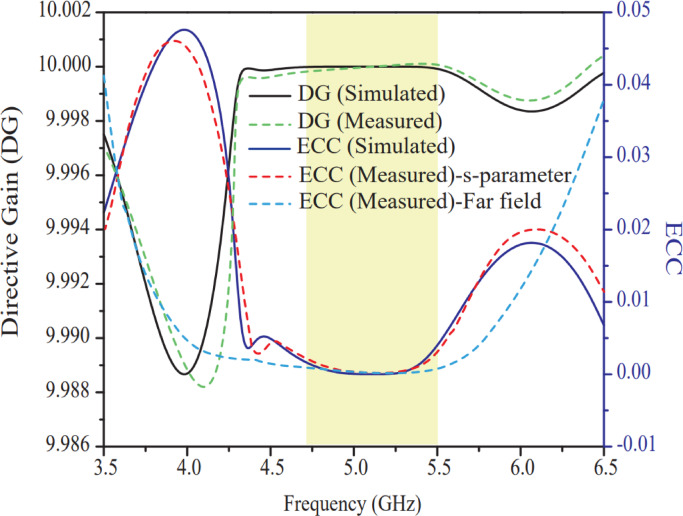
Sim. and meas. ECC and DG plots between port-1 (P1) and port-2 (P2).

The ECC and DG curve of the suggested antenna is illustrated in Fig. 20. Wherein, ECC and DG for ports 1 & 2 study is carried out of 8-ports and other ports are neglected from the study to avoid clustering and same nature of curves from the diagram. In a MIMO arrangement, the ECC study shows how much an antenna element depends on its neighbors when several elements are positioned on the same substrate. The ECC value is a number between 0 and 1, with 0 denoting perfect, total independence between the elements. To ensure that antenna elements behave sufficiently independently, an ECC value of less than 0.5 is generally regarded as appropriate^37^. The ECC curve of the proposed antenna, as shown in Fig. 20, is computed from S-parameters and far-field parameters, with the corresponding expressions provided in Eqs. (10) and (11), respectively. The far-field ECC shows more accurate and reliable results compared to the ECC values computed from S-parameters. However, ECC calculated using S-parameters also provides accurate values when the efficiency is significantly high. In this study, both S-parameter-based ECC and far-field-based ECC exhibit excellent performance, with only a marginal difference across the entire resonating band. For the suggested eight port antenna design, ECC value of ${4.6\times10}^{-6}$ is achieved and the sim. & meas. data of ECC values are found in concurrence. The ECC value of the suggested antenna is substantially less with respect to the antennas^25–34^ as listed in Table 2. In other hand, diversity gain represents how the MIMO antenna performs in multipath fading environment without loss of signal. In practice, 10 dB value of DG is the reference value for efficient MIMO system. The sim. and meas. DG parameter of the suggested antenna is computed using an empirical formula given in Eq. (12). Figure 20 shows approximately 10 dB of DG value which makes the antenna more efficient (improved signal to noise ratio) in fading environment.

10 $${ECC}_{\mathrm{1,2},8}=\frac{{\left|{\sum}_{k=1}^{8}{S}_{k1}^{*}{S}_{k2}\right|}^{2}}{\left(1-{\sum}_{k=1}^{8}{\left|{S}_{k1}\right|}^{2}\right)\left(1-{\sum}_{k=1}^{8}{\left|{S}_{k2}\right|}^{2}\right)}$$

11 $${\mathrm{ECC}}_{\mathrm{Far-field}}=\frac{{\left|{\int}_{4{\uppi}}\left[{E}_{i}\left({\uptheta},{\upvarphi}\right)\cdot{{E}^{*}}_{j}\left({\uptheta},{\upvarphi}\right)\right]\hspace{0.17em}d{\Omega}\right|}^{2}}{\left({\int}_{4{\uppi}}{\left|{E}_{i}\left({\uptheta},{\upvarphi}\right)\right|}^{2}\hspace{0.17em}d{\Omega}\right)\cdot\left({\int}_{4{\uppi}}{\left|{E}_{j}\left({\uptheta},{\upvarphi}\right)\right|}^{2}\hspace{0.17em}d{\Omega}\right)}$$

Where, ${E}_{i}\left({\uptheta},{\upvarphi}\right)$ – complex electric field for *i*^*t*h^ port, ${{E}^{*}}_{j}\left({\uptheta},{\upvarphi}\right)$– complex conjugate electric field for *j*^*t*h^ port, ${\uptheta}$ – elevation angle, ${\upvarphi}$ – azimuth angle, ${\int}_{4{\uppi}}\left(\cdot\right)d{\Omega}$ – integration over solid angle of 4π, $d{\Omega}$ – differential solid angle ($\mathrm{sin}{\uptheta}d{\uptheta}d{\upvarphi}$)

12 $${DG\left(dB\right)}_{\mathrm{1,2},8}=10\sqrt{1-{\left|\left({ECC}_{\mathrm{1,2},8}\right)\right|}^{2}}$$

**Fig. 21:**
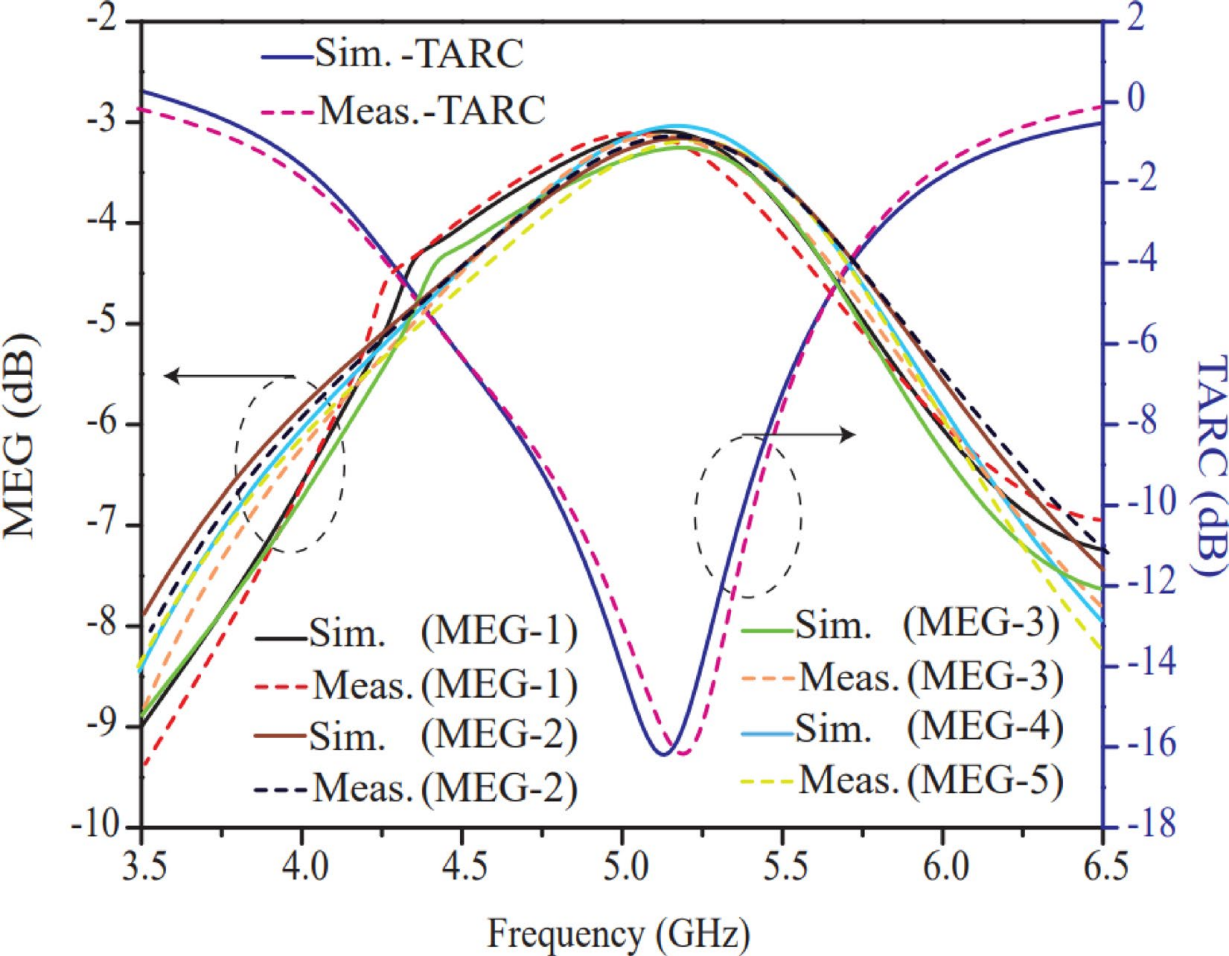
Simulated and measured MEG (at port-1 (P1), port-2 (P2), port-3 (P3), port-4 (P4)) and TARC (between port-1 (P1) and port-2 (P2).) curves.

The TARC & MEG curves of the intended antenna are presented in Fig. 21. Simulated and measured curves of TARC and MEG are computed from Eqs. (9) & (13) which are referred from works as reported in^37,38^. According to published articles^37,38^, less than − 10 dB value for TARC and − 3 < MEG (dB) < −12 value for MEG are in acceptable range. The TARC demonstrates how well the MIMO antenna works if all ports are excited simultaneous with different phases. The TARC curve is more realistic than reflection coefficient curve because it combines all ports whereas reflection coefficient gives information about individual port.

In other hand, MEG defines how well the MIMO antenna is capable to handle the power in fading environment. The suggested antenna offers excellent TARC value in entire operating band and the bandwidth calculated from Figs. 14 and 21 is found to be in close agreement which indicates efficient diversity characteristic of 8 elements MIMO structure. Moreover, the MEG of the proposed antenna varies in −3 to −9 dB range and offers − 3dB (half power) at resonant frequency, which is acceptable range, and it makes antenna suitable for noisy environment where, each element of MIMO antenna will receive at least half of the power. However, four ports (P1–P4) have been considered for the MEG study to avoid clustering and repetition in Fig. 21.

13 $${\mathrm{MEG}}_{{P}_{i}}=0.5\left[1-{\sum}_{j=1}^{8}{\left|{S}_{ij}\right|}^{2}\right]$$

**Fig. 22:**
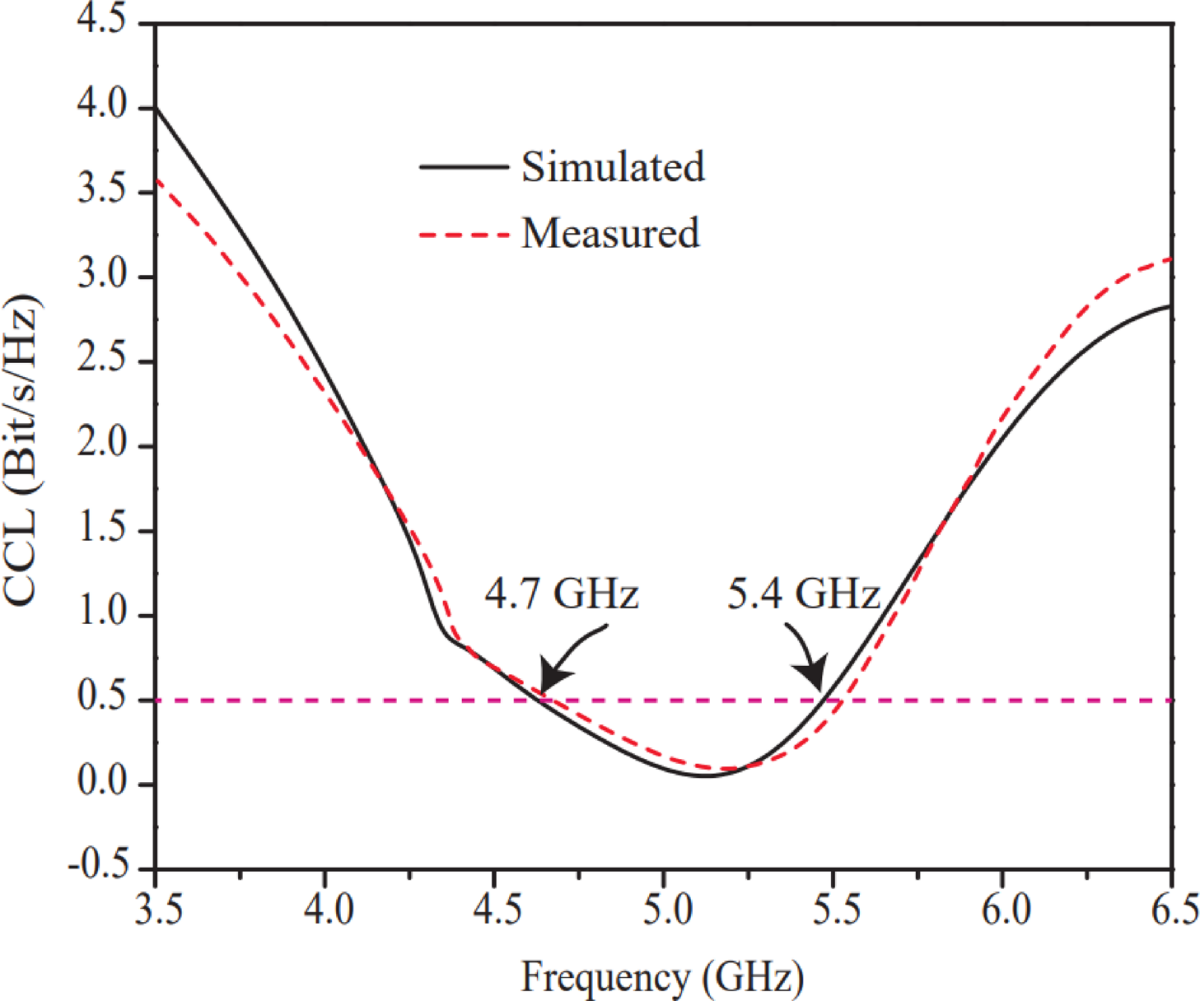
CCL performance curves of the suggested antenna between port-1 (P1) and port-2 (P2).

The CCL curve of the suggested antenna is neatly illustrated in Fig. 22. CCL parameter of MIMO antenna quantifies how much channel capacity is affected by mutual coupling and elements correlation. An ideal value of CCL is 0 dB, but less than 0.4 dB CCL value is considered as in acceptable limit^39^. Simulated and measured CCL curves of the suggested prototype are computed using empirical formulas as given in Eqs. (14)-(17), based on the reported work^39^.

14 $$\mathrm{CCL}=-{\mathrm{log}}_{2}\left(\mathrm{det}\left(\varPsi\right)\right)$$

15 $$\varPsi=\left[\begin{array}{ccc}{\varPsi}_{11}&{\varPsi}_{12}&\begin{array}{cc}\cdots&{\varPsi}_{18}\end{array}\\{\varPsi}_{21}&{\varPsi}_{22}&\begin{array}{cc}\cdots&{\varPsi}_{28}\end{array}\\\begin{array}{c}\vdots\\{\varPsi}_{81}\end{array}&\begin{array}{c}\vdots\\{\varPsi}_{82}\end{array}&\begin{array}{cc}\begin{array}{c}\cdots\\...\end{array}&\begin{array}{c}\vdots\\{\varPsi}_{88}\end{array}\end{array}\end{array}\right]$$

16 $${\varPsi}_{ii}=1-{\sum}_{N=1}^{8}{\left|{\mathrm{S}}_{i,N}\right|}^{2}$$

17 $${\varPsi}_{im}=-\left|{\sum}_{j=1}^{8}{S*}_{ij}{S}_{jm}\right|,1\le\:i\le8,\hspace{0.25em}1\le\:m\le8\text{}$$

The CCL value of the intended antenna is excellent and 0.04 Bit/s/Hz value is achieved at 5.2 GHz resonating frequency. The CCL value (0.04) of the intended antenna is far better with respect to the CCL value of antennas as indicated in Table 2. However, the CCL value of the suggested antenna is greater with respect to the antenna^25^ as mentioned in Table 2 which is 0.02, but antenna^25^ has achieved this value after compromising antenna size, bandwidth, gain, ECC and isolation.

The proposed antenna demonstrates excellent MIMO performance across the entire operating band. In this study, only a few ports have been discussed and illustrated, while the rest have been omitted to avoid clutter and repetition in the figures. Since the performance of each port is approximately identical in nature due to the symmetrical elements and their positioning. Hence, the detailed discussion of every port has been excluded from the discussion.

## Conclusion

A novel, compact and an efficient 8 × 8 MIMO antenna system for 5G NR-n46 and n79 band applications was studied in this article. The performance matrices of the suggested antenna are studied in preceding sections and results are compared with recently published 8-port MIMO antennas^25–34^ for same applications as enumerated in Table 2. After comparison it is seen that the proposed antenna outperforms and established a trade-off between antenna performance parameters. An isolator structure (rectangular ring, centered annular ring and plus shaped slot) on the ground plane serves as to mutual coupling between antenna elements. By altering the ground plane surface current distribution, these etched patterns successfully prevent undesired coupling between nearby radiating elements. The proposed antenna offers a wide band 700 MHz (4.75–5.45 GHz) frequency operation in sub-6 GHz 5G band. The antenna resonates at 5.2 GHz and yields 33 dB isolation, 4.7 dB gain and 92.5% radiation efficiency. MIMO characteristics, such as ECC, DG, TARC, MEG, and CCL, of the proposed antenna were studied and found to be within the permissible range. The high performance of the proposed antenna makes it suitable for Wi-Fi 6 (802.11ax) and Wi-Fi 5 (802.11ac) Routers & Access Points, IoT, Vehicle-to-Everything (V2X) Communication and wireless backhaul systems applications.

## Data Availability

No datasets were generated or analyzed during the current study.
